# Short-term risk of recurrent stroke in symptomatic carotid near-occlusion: pooled analysis of three cohort studies

**DOI:** 10.1093/esj/aakag100

**Published:** 2026-08-24

**Authors:** Andrés García-Pastor, Elias Johansson

**Affiliations:** Vascular Neurology Section and Stroke Centre, Department of Neurology, Hospital General Universitario Gregorio Marañón, Instituto de Investigación Sanitaria Gregorio Marañón (IISGM), Madrid, Spain; Neuroscience and Physiology, Gothenburg University, Gothenburg, Sweden

**Keywords:** carotid near-occlusion, carotid stenosis, full collapse, pooled analysis, stroke recurrence, TIA

## Abstract

**Introduction:**

Carotid near-occlusion (CNO) is a severe carotid stenosis causing distal internal carotid artery (ICA) diameter reduction, classifiable as with or without full collapse. Whether full collapse confers an especially high short-term recurrent stroke risk is debated, and no prior pooled analysis has quantified the ultra-early (0–2 day) risk.

**Patients and methods:**

Pooled individual patient data from 3 prospective cohort studies (TransAtlantic Carotid Near-Occlusion Study [TACNOS], UCC [Umeå Carotid Cohort], CAsi Oclusión Sintomática [CAOS]). The primary outcome was ipsilateral ischaemic stroke or retinal infarction within 90 days, censored at revascularisation. Full collapse was defined by visual appearance and by measurement (distal ICA ≤ 2.0 mm and/or ICA ratio ≤ 0.42). Cox regression and pre-specified sensitivity analyses addressing ascertainment bias and informative censoring were performed.

**Results:**

A total of 360 patients with symptomatic CNO were included. The 90-day recurrent stroke risk was 16.4% (95% CI, 11.7%–21.1%), with 5.9% within the first 2 days. Per-cohort rates differed markedly (TACNOS 23.9%, UCC 22.3%, CAOS 8.1%; *I*^2^ = 77%, *P* = .012). By visual appearance (*n* = 333), 90-day stroke risk did not differ by collapse status (adjHR 1.5; 95% CI, 0.8–2.9). By measurement (*n* = 219), full collapse was associated with higher stroke risk (adjHR 2.0; 95% CI, 1.0–3.9). For both definitions, full collapse strongly predicted 0–2 day recurrence (adjHR 4.0; 95% CI, 1.5–10.5 and adjHR 4.8; 95% CI, 1.8–12.8). Full collapse combined with TIA identified a subgroup with 90-day stroke risk of 37.8% (by appearance) and 50.4% (by measurement).

**Discussion:**

Findings should be interpreted in the context of substantial between-study heterogeneity and informative censoring; sensitivity analyses confirmed the robustness of the estimates.

**Conclusions:**

Carotid near-occlusion with full collapse carries a markedly elevated risk of stroke recurrence within 48 h, supporting dedicated trials of urgent revascularisation strategies. In exploratory analysis, presentation with TIA appeared to identify a particularly high-risk subgroup, a hypothesis-generating observation that requires independent validation.

## Graphical Abstract

**Graphical Abstract:**
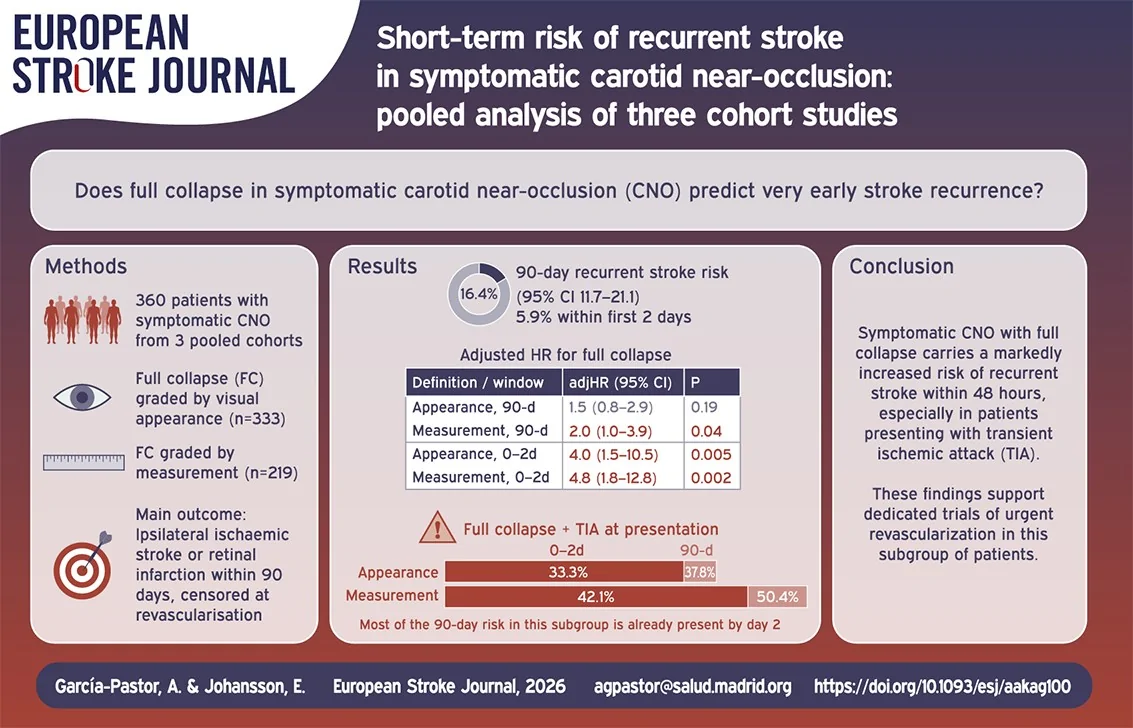

## Introduction

**Figure 1:**
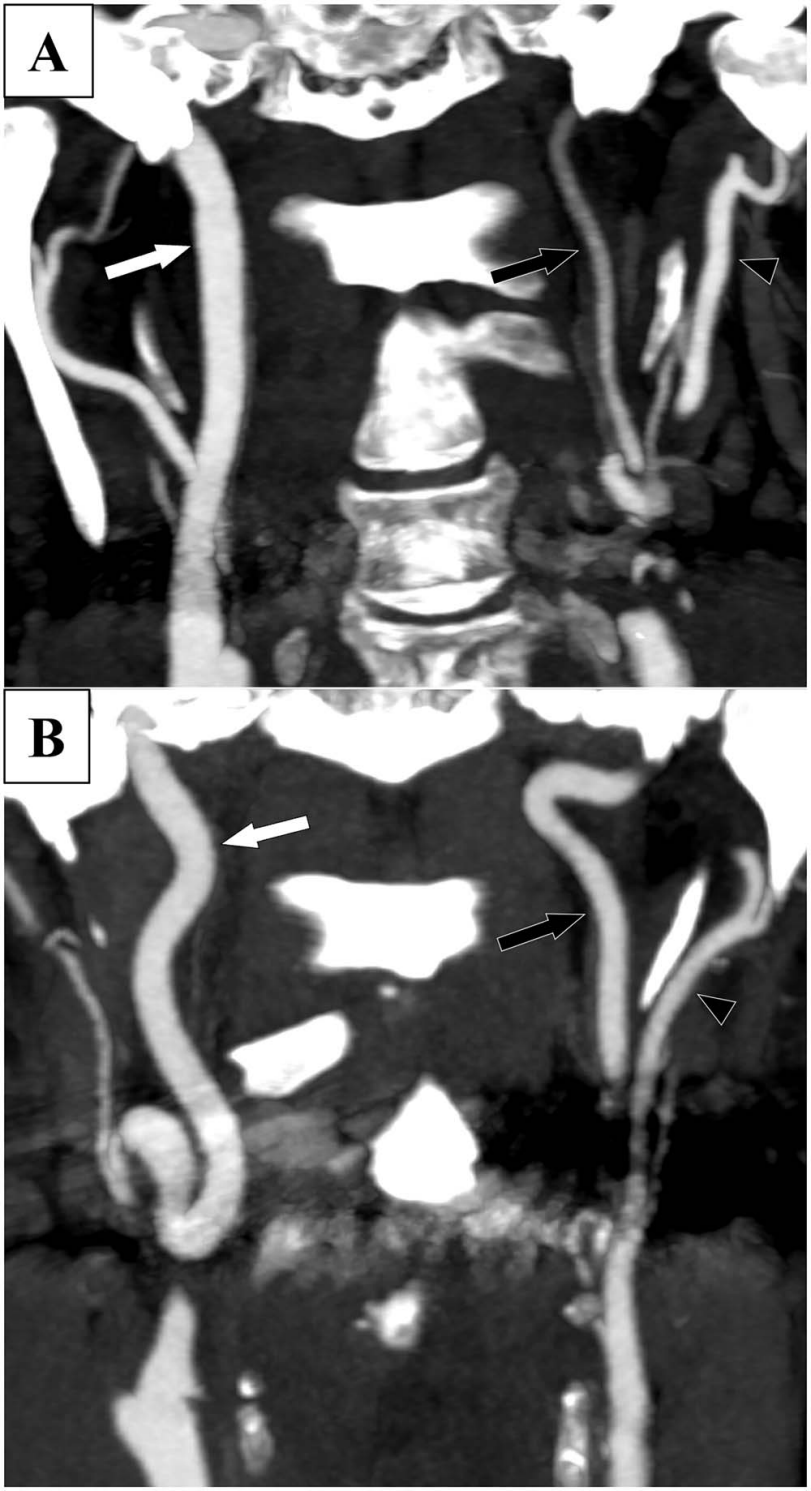
Two examples of left-sided carotid near-occlusion on CTA, coronal views. (A) With full collapse. Distal to a severe stenosis (not in plane), the distal ICA is very small (1.1 mm diameter, white arrow), clearly smaller than the contralateral ICA (5.4 mm, black arrow) and the ipsilateral ECA (2.4 mm, arrowhead). (B) Without full collapse. Distal to a severe stenosis (not in plane), the distal ICA is small (2.9 mm diameter, white arrow), clearly smaller than the contralateral ICA (4.6 mm, black arrow) and of similar diameter to the ipsilateral ECA (2.5 mm, arrowhead). Abbreviations: ECA = external carotid artery; ICA = internal carotid artery.

Carotid near-occlusion (CNO) is defined as a very tight stenosis in which the diameter of the internal carotid artery (ICA) beyond the stenosis is reduced.^1^^,^^2^ Only stenoses not causing distal ICA collapse (conventional stenoses) are graded with percent using the North American Symptomatic Carotid Endarterectomy Trial (NASCET) grading.^1^ Diagnostic separation of CNO from conventional stenosis is challenging, as overlaps with anatomical variants preclude the use of simple size thresholds; diagnosis instead relies on assessment of multiple features, including severe stenosis, small distal ICA in absolute terms and relative to the contralateral ICA and ipsilateral external carotid artery and haemodynamic measurements.^3^ Carotid near-occlusion can be subdivided into cases with severe distal ICA collapse (full collapse, previously termed “string sign”; Figure 1A) and those in which the distal ICA diameter is reduced while the vessel remains visually normal in appearance (without full collapse; Figure 1B).^1^ Separation of these subtypes is achieved either by visual appearance or by measurement criteria.^4^

Carotid endarterectomy (CEA) of symptomatic CNO showed no net benefit in the pooled analysis of NASCET and the European Carotid Surgery Trial (ECST), as the medical arms had a relatively favourable prognosis.^2^ Subsequent studies have suggested a possible benefit of revascularisation in patients with CNO,^5^ and recent reports indicate that CNO with full collapse carries a high short-term risk of recurrent ipsilateral ischaemic stroke,^6–8^ although not all studies have confirmed this finding.^9^ North American Symptomatic Carotid Endarterectomy Trial and ECST included very few cases with full collapse and did not assess early recurrence risk.^2^ Three recent international guidelines address CNO differently: the 2022 Society of Vascular Surgery guidelines barely mention CNO, the 2021 European Stroke Organisation guidelines offer no management recommendation, and the 2023 European Society for Vascular Surgery (ESVS) guidelines provide extensive discussion.^10–12^ The ESVS guidelines recommend revascularisation (CEA or carotid artery stenting [CAS]) only after multidisciplinary review in cases with recurrent symptoms despite best medical therapy, without incorporating full collapse status into the recommendation.^12^

These ESVS guidelines are in part based on an individual patient-data meta-analysis (IPDMA) where the risk of stroke with medical treatment, CEA or CAS in patients with symptomatic CNO was summarised.^5^ That IPDMA did not assess if included studies diagnostics were reasonably similar to that of NASCET and ECST, did not focus on analysing the risk of recurrence before revascularisation (the emphasis was on comparing managements) and was not able to compare with and without full collapse.^5^ Following the publication of that study, 2 studies about short-term stroke risk for symptomatic CNO have been presented, substantially increasing the number of published cases.^7^^,^^8^ There are calls for additional assessments of prognosis in symptomatic CNO.^12^^,^^13^

Three evidence gaps motivate the present analysis. First, no prior pooled study has systematically quantified the ultra-early (0–2 day) recurrent stroke risk in symptomatic CNO, the critical window within which any preventive intervention must operate. Second, the effect of full collapse on early recurrence risk has not been formally assessed in a pooled analysis incorporating all available data. Third, identification of clinical subgroups at particularly elevated early risk is required to define the target population for future randomised trials. The present analysis addresses these gaps by pooling individual patient data from all eligible cohort studies, with emphasis on the comparison between patients with and without full collapse.

## Patients and methods

### Systematic search

A previously performed systematic search^1^ was updated on June 5, 2024. PubMed was searched using the terms “carotid near-occlusion,” “carotid pseudo-occlusion,” “carotid string sign,” “carotid slim sign,” “carotid critical stenosis,” “small distal carotid artery,” “narrow distal carotid artery,” “carotid preocclusive stenosis,” “carotid pre-occlusive stenosis,” “carotid subtotal stenosis,” “carotid subtotal occlusion,” “carotid functional occlusion,” “carotid sub-occlusion,” “carotid hypoplasia,” “carotid incomplete occlusion” and “carotid hairline” from January 2015 to June 2024. The term “carotid near total occlusion” was added without time restrictions. Reference lists of all identified studies assessing CNO in any fashion (*n* = 144) were also screened.

### Study selection

Studies reporting recurrent stroke risk before and/or without revascularisation in patients with symptomatic CNO were selected if they fulfilled all of the following criteria: (1) inclusion of ≥ 20 patients with CNO; (2) use of the presenting event as the index time point, or availability of data allowing such analysis; (3) reporting of full collapse status or availability of the underlying data and (4) diagnosis of CNO according to NASCET criteria^2^ or equivalent criteria in which distal ICA calibre reduction was the key diagnostic feature, without restricting the CNO definition to cases with full collapse. The threshold of ≥ 20 cases was selected as the minimum required to generate Kaplan–Meier event-rate estimates with sufficiently narrow confidence intervals and Cox regression models with adequate statistical power. Studies based solely on ultrasound were excluded, as CNO can present with any stenosis velocity on carotid ultrasound.^14–16^ Low stenosis velocity without angiographic confirmation was not accepted as a diagnostic criterion, as it identifies only approximately 20% of cases with CNO.^1^

Full collapse was defined by appearance when a thread-like lumen was identified distal to the stenosis, and by measurement when the distal ICA diameter was ≤ 2.0 mm and/or the ICA ratio was ≤ 0.42.^4^ In all cohorts, CNO was identified by feature interpretation—a severe stenosis with a reduced distal ICA calibre, assessed in absolute terms and relative to the contralateral ICA and the ipsilateral ECA—which distinguishes it from conventional ≥ 50% stenosis graded by NASCET percent. The diagnostic methods used in each included study are detailed in the online supplement. Authors of eligible studies were contacted, and individual patient data were pooled into a single database.

### Data harmonisation

Details are provided in the online-only supplemental material.

### Risk of bias

Risk of bias was assessed using the Newcastle–Ottawa Scale for cohort studies.^17^

### Outcome definition

The primary outcome was recurrent ipsilateral ischaemic stroke or retinal infarction (hereafter “recurrent stroke”) within 90 days of the presenting event. Stroke required symptom duration > 24 h, ie, clinical TIA with radiological evidence of infarction was not considered as a stroke. In patients undergoing revascularisation within 90 days, only recurrences preceding the procedure were counted.

### Statistical analysis

Data are presented with 95% CIs. Normally distributed continuous variables are expressed as mean and SD; between-group comparisons used one-way ANOVA. Non-normally distributed continuous variables are expressed as median and IQR, with comparisons using the Kruskal–Wallis test. Categorical variables are expressed as percentages, with comparisons using the 2-sided χ^2^ test.

Time-to-event analyses were performed using Kaplan–Meier estimation (with log-rank test) and Cox proportional hazards regression. Revascularisation, death, loss to follow-up and reaching 90 days served as censoring events. Covariates associated with the outcome at *P* < .10 in univariable Cox regression were entered into multivariable models. Given the collinearity between the 2 full collapse definitions (95% agreement in cases with data for both), appearance-based and measurement-based analyses were conducted separately. To allow a like-for-like comparison of the 2 definitions in a common population—the measurement definition being available only in TransAtlantic Carotid Near-Occlusion Study (TACNOS) and Umeå Carotid Cohort (UCC)—the pre-specified appearance-based analysis restricted to TACNOS and UCC (*n* = 219) is reported alongside the measurement-based analysis.

An exploratory multivariable Cox model analysis adjusting for age, hypertension, diabetes and previous stroke or TIA was fitted for each full-collapse definition and time window. This model is exploratory and hypothesis-generating given the limited number of events.

As log–log plots indicated that full collapse was borderline non-compliant with the proportional hazards assumption, analyses were additionally stratified into the periods 0–2 days and 3–90 days. Between-study heterogeneity for the 90-day and 0–2 day Kaplan–Meier estimates was quantified by random-effects meta-analysis using the DerSimonian–Laird estimator applied to logit-transformed cumulative incidence; an *I*^2^ statistic > 50% was considered indicative of substantial heterogeneity.

As 53% of patients underwent revascularisation within 90 days, constituting a form of informative censoring (more unstable patients may have been prioritised for earlier intervention), observed recurrence rates under the primary analysis should be interpreted as conservative (minimum) estimates. To assess the impact of this censoring structure, an Aalen–Johansen cumulative incidence function (CIF) estimator treating revascularisation as a competing event was computed in addition to the primary Cox model; results are reported in the online supplement.

Pre-specified sensitivity analyses comprised: (1) exclusion of cases in which recurrent stroke had triggered the CNO diagnosis (ascertainment bias analysis, detailed in Table S11); and (2) restriction of the appearance-based analysis to the TACNOS and UCC cohorts only, for direct comparison with the measurement-based analysis. A 2-sided *P*-value < .05 was considered statistically significant. Analyses were performed using IBM SPSS version 28.0 (IBM Corp., Armonk, NY), Jamovi version 2.3.28.0 and Python version 3.11 with the lifelines library (Aalen–Johansen estimator implementation).

## Results

### Search results and study selection

The systematic search yielded 801 articles, of which 144 assessed at least one aspect of CNO (Figure S1). Reference list searches identified 10 additional articles, and 1 further article was identified through personal communication. Of the 144 articles, 14 assessed stroke recurrence risk without revascularisation; of these, 3 studies met all eligibility criteria^7–9^ (Figure S1).

### Risk of bias assessment

Newcastle–Ottawa Scale scores were 8 for CAOS (CAsi Oclusión Sintomática) and 9 for both TACNOS and UCC, indicating a low risk of bias across all 3 studies.

### Baseline characteristics

A total of 360 patients were included: TACNOS (*n* = 101), UCC (*n* = 118) and CAOS (*n* = 141). Statistically significant differences between cohorts were observed for nearly all baseline characteristics (Table 1), including the proportion presenting with TIA, the proportion with full collapse and the median time from presenting event to angiography (2–4 days in TACNOS and UCC vs 12 days in CAOS).

**Table 1:** Baseline characteristics of the 3 included cohorts.

	All (*n* = 360)	TACNOS (*n* = 101)	UCC (*n* = 118)	CAOS (*n* = 141)	*P* ^a^
**Study site**	–	Umeå, Sweden	Umeå, Sweden	Several, Spain	–
**Study years**	–	2010-2014	2018-2022	2010-2016	–
**Age, years, mean (SD)**	71 (9)	70 (9)	74 (7)	69 (9)	<.001
**Women, *n* (%)**	87 (24)	31 (31)	35 (30)	21 (15)	.004
**Ischaemic heart disease^b^, *n* (%)**	86 (24)	30 (30)	33 (28)	23 (16)	.025
**Symptomatic peripheral artery disease, *n* (%)**	29 (8)	9 (9)	7 (6)	13 (9)	.58
**Any stroke or TIA > 6 months before presenting event^c^, *n* (%)**	84 (23)	28 (28)	23 (19)	33 (23)	.357
**Atrial fibrillation, *n* (%)**	29 (8)	10 (10)	12 (10)	7 (5)	.24
**Diabetes, *n* (%)**	101 (28)	22 (22)	28 (24)	51 (36)	.022
**Dyslipidaemia^d^, *n* (%)**	256 (71)	72 (71)	111 (94)	73 (52)	<.001
**Current smoker, *n* (%)**	96 (27)	24 (24)	23 (20)	49 (35)	.016
**Hypertension^e^, *n* (%)**	286 (79)	85 (84)	100 (85)	101 (72)	.013
**Recent ipsilateral ischaemic event ≤ 7 days before presenting event^f^, *n* (%)**	61 (17)	15 (15)	27 (23)	19 (13)	.107
**Presenting event: retinal, *n* (%)**	67 (19)	16 (16)	40 (34)	11 (8)	<.001
**Presenting event: TIA, *n* (%)**	104 (29)	36 (36)	34 (29)	34 (24)	
**Presenting event: stroke, *n* (%)**	189 (53)	49 (49)	44 (37)	96 (68)	
**Days between presenting event and angiography^g^ median (IQR)**	5 (1–15)	4 (1–12)	2 (0–9)	12 (6–21)	<.001
**Angiography type**	–	CTA	CTA	CA	–
**Full collapse by visual appearance^h^**	107 (32)	43 (43)	23 (20)	41 (36)	<.001
**Full collapse by CTA measurement^i^**	66 (30)	40 (40)	26 (22)	NA	.005
**Underwent revascularization within 90 days, *n* (%)**	190 (53)	50 (50)	82 (70)	58 (41)	<.001
**CEA within 90 days, *n* (%)**	133 (70)	50 (100)	65 (79)	18 (31)	<.001
**CAS within 90 days, *n* (%)**	57 (30)	0 (0)	17 (21)	40 (69)	
**Days between presenting event revascularization median (IQR)^j^**	13 (8–31)	12 (7–24)	10 (6–21)	24 (11–51)	<.001
**Any stroke or death < 30 days of revascularization, *n* (%)**	9 (3)	2 (4)	3 (4)	4 (3)	1.0
**Follow-up in main analysis, days, median (IQR)**	25 (9–90)	18 (6–90)	12 (6–46)	90 (20–90)	<.001

Abbreviations: CA = conventional angiography; CAOS = CAsi Oclusión Síntomatica; CAS = carotid stenting; CEA = carotid endarterectomy; NA = not available; RAO = retinal artery occlusion; TACNOS = TransAtlantic Carotid Near-Occlusion Study; UCC = Umeå Carotid Cohort.

^a^2-sided χ^2^ test for categorical, one-way-ANOVA for continuous data presented as mean, Kruskal–Wallis for continuous data presented as median.

^b^Previous myocardial infarction, current angina and/or previous coronary intervention.

^c^Any ischaemic stroke or TIA occurring more than 6 months before the presenting event (remote cerebrovascular history).

^d^Total cholesterol > 200 mg/dL, Low-density lipoprotein > 120 mg/dL and/or lipid-lowering medication.

^e^Systolic pressure > 140 mmHg, diastolic pressure > 90 mmHg and/or use of blood-pressure reducing medication.

^f^Ipsilateral ischaemic stroke, TIA or retinal event referable to the near-occluded artery within 7 days before the qualifying presenting event (recent/crescendo ipsilateral symptoms).

^g^Missing data in 36 cases in CAOS.

^h^Missing data in 27 cases in CAOS.

^i^Missing data in 27 cases in CAOS.

^j^No patient with full collapse (by either definition) underwent CEA or CAS within 48 h of presentation, except for 3 cases in UCC who underwent CAS as part of thrombectomy for the presenting event.

In the 2 Swedish cohorts (TACNOS and UCC, single tertiary centre), CNO was identified from CTA obtained during the standard work-up of patients with suspected anterior-circulation TIA or ischaemic stroke—retrospectively from consecutive CTA examinations in TACNOS and prospectively in UCC. In the CAOS multicentre registry, patients with suspected symptomatic carotid disease were screened (usually with ultrasound and/or CTA) and those with suspected near-occlusion underwent confirmatory conventional angiography.

In the 3 cohorts, medical prevention was initiated as early as possible. The standard treatment was a combination of antiplatelets and statins, with blood pressure reduction and antidiabetic treatment as needed. While exact medication data is not available, the usual treatment was single antiplatelet therapy. Dual antiplatelet therapy was foremost used in cases undergoing CAS, but due to emerging evidence became more common also without stenting in the final years of the data collection.

Peri-procedural stroke or death rates within 30 days of revascularisation in patients who underwent CEA or CAS are presented in Table S1.

### Ninety-day risk of recurrent stroke

Of the 360 patients, 46 experienced a recurrent stroke, yielding a 90-day cumulative risk of 16.4% (95% CI, 11.7%–21.1%); 21 events (5.9%; 95% CI, 3.4%–8.3%) occurred within the first 2 days. Per-cohort 90-day risks differed markedly: TACNOS 23.9% (95% CI, 15.6%–35.5%), UCC 22.3% (13.7%–35.1%) and CAOS 8.1% (4.2%–15.2%) (Table S2). Between-study heterogeneity for the 90-day risk was substantial (*I*^2^ = 77.4%, *Q* = 8.85, *P* = .012). The corresponding 0–2 day risks displayed an even steeper gradient: TACNOS 11.9% (6.9%–20.0%), UCC 6.1% (2.9%–12.3%) and CAOS 1.4% (0.4%–5.6%) (*I*^2^ = 77.3%, *Q* = 8.80, *P* = .012). Subgroup analyses for all cases are presented in Figure S2.

**Figure 2:**
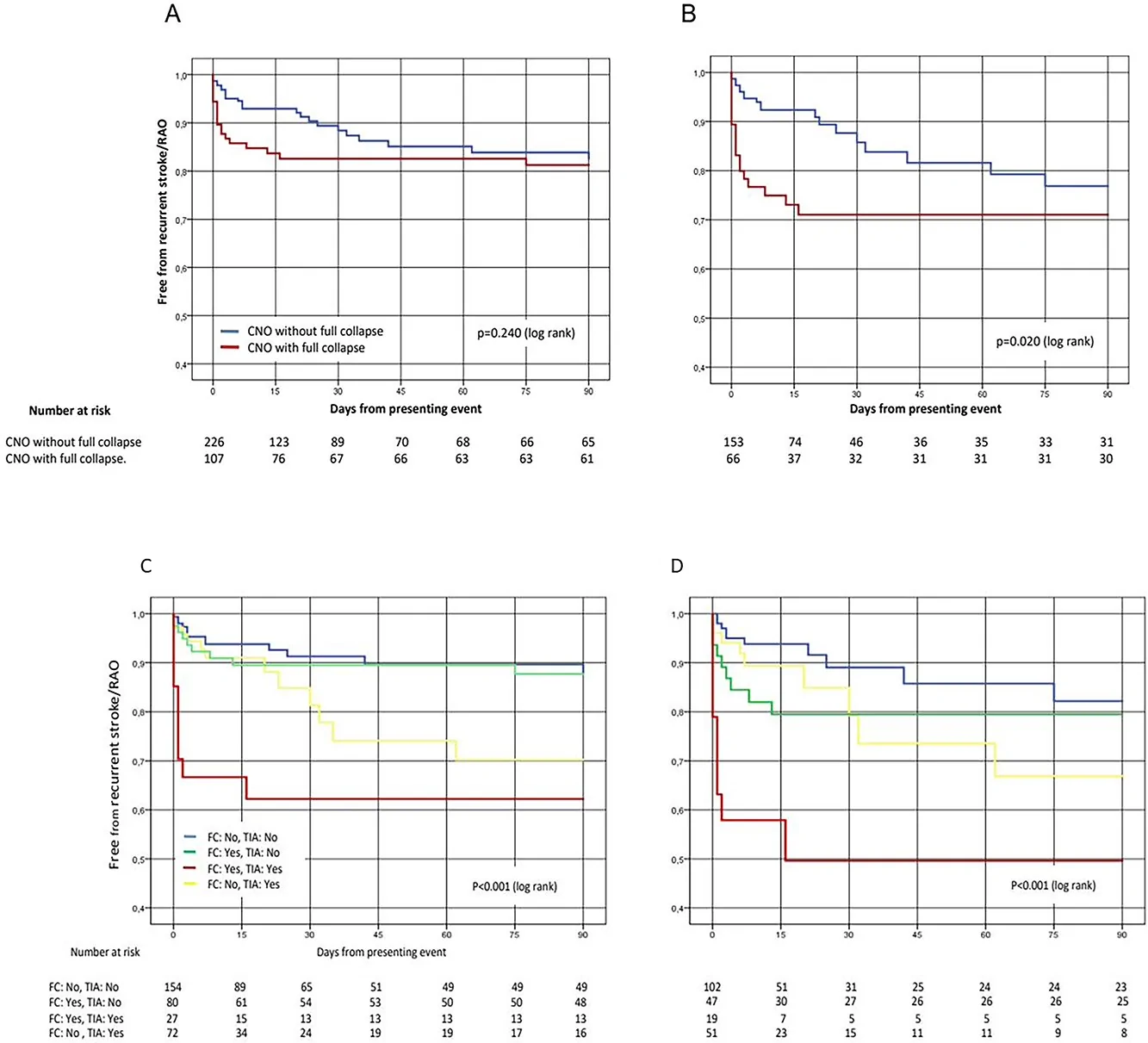
Kaplan–Meier estimates of cumulative risk of recurrent stroke within 90 days of the presenting event. (A) Full collapse defined by visual appearance. (B) Full collapse defined by measurement criteria (distal internal carotid artery diameter ≤ 2.0 mm and/or ICA ratio ≤ 0.42). (C) and (D) Kaplan–Meier curves assessing the risk of recurrent stroke within 90 days of presenting event by combinations of full collapse and TIA as presenting event. (C) Full collapse defined by appearance. (D) Full collapse defined by measurement. Abbreviation: ICA = internal carotid artery.

**Figure 3:**
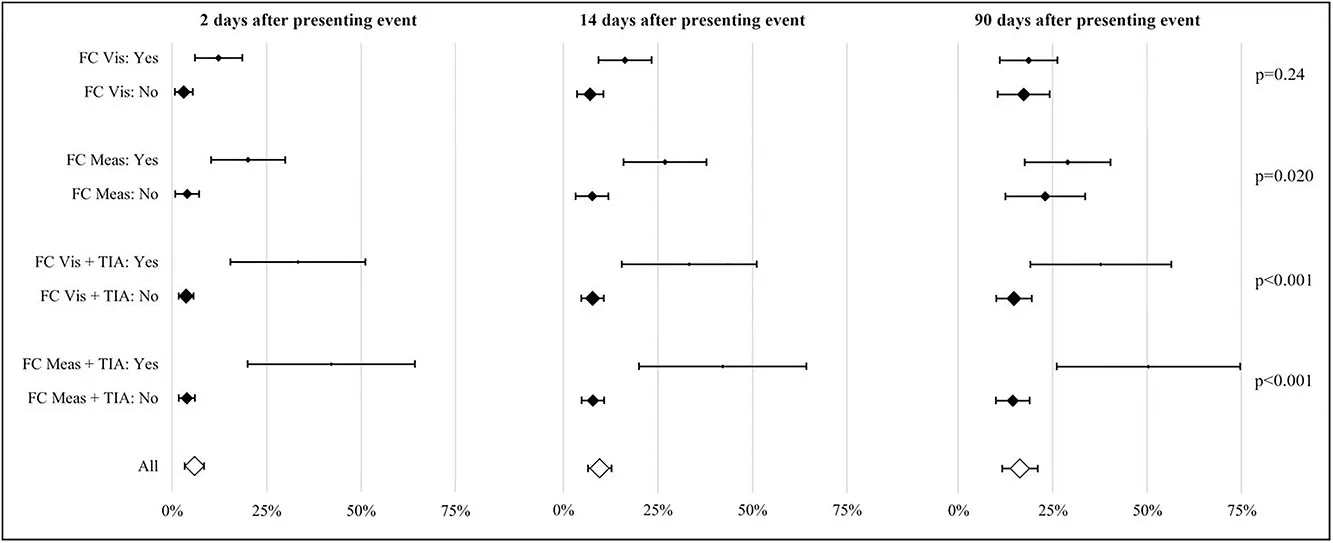
Forest plots of risk of Kaplan–Meier assessments of risk of recurrent stroke within 90 days of presenting event in subgroups that includes full collapse status. Abbreviations: FC Meas = full collapse defined by measurement; FC Vis = full collapse defined by appearance.

Full collapse defined by appearance was available in 333 (93%) patients. The 90-day risk of recurrent stroke did not differ significantly between patients with and without full collapse (18.7%; 95% CI, 11.1%–26.3% vs 17.4%; 95% CI, 10.5%–24.3%; log-rank *P* = .24; Figure 2A; Figure 3; Table S3). Cohort and TIA as presenting event affected recurrence risk; after adjustment for these factors, full collapse did not reach statistical significance (adjHR 1.5; 95% CI, 0.8–2.9; *P* = .19; Table 2; Table S4).

**Table 2:** Multivariable analysis: hazard ratios for recurrent stroke within 90 days of the presenting event.

	Bivariable HR (95%CI)	*P*	Model 1		Model 2	
			Multivariable HR (95%CI)	*P*	Multivariable HR (95%CI)	*P*
**Full collapse defined by appearance, *n* = 333, 44 recurrent strokes**						
** Full collapse**	1.4 (0.8–2.6)	.25	1.5 (0.8–2.9)	.190	Not used	–
** Cohort: CAOS**	Ref	–	Ref	–	Ref	–
** Cohort: TACNOS**	3.9 (1.6–9.3)	.002	3.5 (1.5–8.4)	.005	3.6 (1.5–8.7)	.004
** Cohort: UCC**	3.1 (1.3–7.5)	.012	3.9 (1.5–9.6)	.004	3.5 (1.4–8.5)	.007
** Women**	2.0 (1.1–3.6)	.030	1.4 (0.8–2.6)	.27	1.5 (0.8–2.8)	.22
** Presenting event: retinal**	0.4 (0.1–1.5)	.17	0.3 (0.1–1.2)	.095	Not used	–
** Presenting event: stroke**	Ref	–	Ref	–	Ref	–
** Presenting event: TIA^a^**	2.3 (1.2–4.2)	.009	2.1 (1.1–4.0)	.024	Not used	–
** Full collapse and presenting event TIA**	3.7 (1.8–7.4)	<.001	Not used	–	3.8 (1.8–7.9)	<.001
**Full collapse defined by measurement, *n* = 219, 27 recurrent strokes**						
** Full collapse**	2.1 (1.1–4.1)	.024	2.0 (1.0–3.9)	.040	Not used	–
** Presenting event: stroke**	Ref	–	Ref	–	Ref	–
** Presenting event: retinal**	0.2 (0.04–0.8)	.025	0.2 (0.1–1.0)	.049	Not used	–
** Presenting event: TIA^b^**	1.8 (0.8–3.1)	.18	1.8 (0.9–3.6)	.082	Not used	–
** Full collapse and presenting event TIA**	4.3 (2.0–9.1)	<.001	Not used	–	4.3 (2.0–9.1)	<.001

Abbreviations: CAOS = CAsi Oclusión Síntomatica; HR = hazard ratio; TACNOS = TransAtlantic Carotid Near-Occlusion Study; UCC = Umeå Carotid Cohort.

Two separate analyses, 1 for the population of 333 cases with data on full collapse by visual assessment, 1 for the population of 219 cases with full collapse by measurement assessment. Co-variates with a possible association (*P* < .10) with outcome in each of the 2 populations were included in the multivariable models. Model 1: standard approach. Model 2: merging presenting event and full collapse and controlling for remaining co-variates. Co-variates not included in either multivariable model (due to *P* > 0.1 in the bivariable analysis) are presented in Table S4.

^a^When using non-TIA events as reference, HR 2.7 (1.5–4.9), *P* = .001 in bivariable and HR 2.6 (1.4–4.9), *P* = .002 in model 1. TIA (vs non-TIA) and full collapse did not have an interaction: *P* = .28.

^b^When using non-TIA events as reference, HR 2.3 (1.2–4.4), *P* = .011 in bivariable and HR 2.5 (1.3–4.9), *P* = .005 in model 1. TIA (vs non-TIA) and full collapse did not have an interaction: *P* = .36.

Full collapse defined by measurement was available in 219 (61%) patients, comprising all TACNOS and UCC cases but none from CAOS. In this subgroup, the 90-day recurrent stroke risk was significantly higher in patients with full collapse than in those without (29.0%, 95% CI, 17.6%–40.4% vs 23.1%; 95% CI, 12.5%–33.7%; log-rank *P* = .020; Figure 2B; Figure 3; Table S3). After adjustment for retinal presenting event, full collapse remained independently associated with recurrent stroke (adjHR 2.0; 95% CI, 1.0–3.9; *P* = .04; Table 2; Table S4).

When the appearance-based analysis was restricted to the same TACNOS + UCC population as the measurement-based analysis (*n* = 219), the 2 definitions yielded concordant estimates: adjHR 4.9 (1.9–12.7), *P* < .001 for the 0–2 day risk and 1.8 (0.9–3.5), *P* = .084 in the 90-day analysis (Table S5).

### Risk of recurrent stroke at 0–2 days and 3–90 days

For both definitions, the 0–2 day risk of recurrent stroke was significantly higher among patients with full collapse: 12.3% (95% CI, 6.0%–18.3%) vs 3.1% (95% CI, 0.7%–5.5%) by appearance and 20.1% (95% CI, 10.3%–29.9%) vs 4.0% (95% CI, 0.9%–7.1%) by measurement (*P* ≤ .001; Table S3). These associations persisted after multivariable adjustment (adjHR 4.0; 95% CI, 1.5–10.5 and adjHR 4.8; 95% CI, 1.8–12.8; *P* ≤ .005; Table 3; Table S6). Neither definition of full collapse was significantly associated with recurrent stroke during days 3–90 (Tables S3 and S7).

**Table 3:** Multivariable analysis: hazard ratios for recurrent stroke within 0–2 days of the presenting event.

	Bivariable HR (95% CI)	*P*	Model 1		Model 2	
			Multivariable HR (95% CI)	*P*	Multivariable HR (95% CI)	*P*
**Full collapse defined by appearance, *n* = 333, 20 recurrent strokes**						
** Full collapse**	4.1 (1.6–10.2)	.003	4.0 (1.5–10.5)	.005	Not used	–
** Cohort: CAOS**	Ref	–	Ref	–	Ref	–
** Cohort: TACNOS**	13.9 (1.8–106.9)	.011	12.8 (1.6–99.7)	.015	13.1 (1.7–102.9)	.015
** Cohort: UCC**	6.9 (0.9–56.2)	.070	10.4 (1.3–86.4)	.030	9.3 (1.1–76.9)	.040
** Women**	3.8 (1.6–9.1)	.003	2.4 (1.0–6.0)	.051	2.5 (1.0–6.0)	.049
** Atrial fibrillation**	2.8 (0.9–8.2)	.070	2.4 (0.8–7.6)	.13	2.1 (0.7–6.5)	.20
** Presenting event: stroke**	Ref	–	Ref	–	Ref	–
** Presenting event: retinal**	0.0 (0.0–> 100)	.96	0.0 (0.0–> 100)	.96	Not used	–
** Presenting event: TIA^a^**	2.6 (1.1–6.3)	.038	2.6 (1.0–6.4)	.047	Not used	–
** Full collapse and presenting event TIA**	10.3 (4.2–24.8)	<.001	Not used	–	8.5 (3.3–21.8)	<.001
**Full collapse defined by measurement, *n* = 219, 19 recurrent strokes**						
** Full collapse**	5.5 (2.1–14.3)	<.001	4.8 (1.8–12.8)	.002	Not used	–
** Women**	2.8 (1.1–6.8)	.026	2.1 (0.9–5.3)	.11	2.0 (0.8–5.1)	.14
** Presenting event: stroke**	Ref	–	Ref	–	Ref	–
** Presenting event: retinal**	0.0 (0.0–> 100)	.95	Not used	–	Not used	–
** Presenting event: TIA^b^**	1.8 (0.7–4.6)	.19	Not used	–	Not used	–
** Full collapse and presenting event TIA**	8.8 (3.5–22.0)	<.001	Not used	–	7.4 (2.9–19.1)	<.001

Abbreviations: CAOS = CAsi Oclusión Síntomatica; HR = hazard ratio; TACNOS = TransAtlantic Carotid Near-Occlusion Study; UCC = Umeå Carotid Cohort.

Same analysis approach and models as in Table 2. Co-variates not included in either multivariable model (due to *P* > .1 in the bivariable analysis) are presented in Table S6.

^a^When using non-TIA events as reference, HR 3.6 (1.5–8.8), *P* = .005 in bivariable. If TIA vs non-TIA was entered in model 1, full collapse was HR 4.4 (1.7–11.7), *P* = .003, TACNOS 12.2 (1.6–96.1), *P* = .017, UCC 8.9 (1.1–74.2), *P* = .043, TIA HR 3.5 (1.4–8.7), *P* = .008, women 2.5 (1.0–6.0), *P* = .050 and atrial fibrillation HR 2.3 (0.7–7.3), *P* = .16. TIA (vs non-TIA) and full collapse did not have an interaction: *P* = .12.

^b^When using non-TIA events as reference, HR 3.0 (1.2–7.5), *P* = .018 in bivariable. If TIA vs non-TIA was entered in model 1, full collapse was HR 5.3 (2.0–14.2), *P* < .001, TIA HR 3.2 (1.3–8.0), *P* = .014 and women HR 1.9 (0.7–4.7), *P* = .19. TIA (vs non-TIA) and full collapse did not have an interaction: *P* = .44.

In an exploratory model additionally adjusting for age, hypertension, diabetes and previous stroke or TIA, the association between full collapse and recurrent stroke was essentially unchanged (0–2 day adjusted HR 4.1 [95% CI, 1.6–10.5] by appearance and 5.2 [1.9–13.9] by measurement; 90-day adjusted HR 2.1 [1.1–4.1] by measurement), and none of these comorbidities was independently associated with recurrence (Table S8).

### Combined analysis: full collapse and TIA as presenting event

Transient ischemic attack as presenting event was independently associated with a higher risk of recurrent stroke in bivariable and multivariable analyses (Figure S3; Tables 2 and 3; Table S9). A combined variable of full collapse and TIA was associated with a markedly elevated 90-day recurrent stroke risk: 37.8% (95% CI, 19.2%–56.4%) for the appearance definition and 50.4% (95% CI, 26.1%–74.6%) for the measurement definition (Tables 2 and 3; Figure 2C and D; Tables S9 and S10). The great majority of these recurrences occurred within the first 2 days.

### Sensitivity analyses

The ascertainment bias sensitivity analysis, in which the recurrent stroke was treated as the index event for cases in which stroke triggered the CNO diagnosis (*n* = 7, all without full collapse; details in Table S11), yielded a 90-day recurrence risk of 10.8% for CNO without full collapse, lower than the primary estimate of 17.4%, while the full collapse group estimate remained unchanged at 18.7%, resulting in a statistically significant between-group difference (*P* = .027). The competing risks sensitivity analysis treating revascularisation as a competing event yielded CIF estimates that were modestly lower than the corresponding Kaplan–Meier estimates, as expected; the relative effect of full collapse was preserved (90-day CIF: 17.8% with full collapse vs 11.1% without, by appearance; 27.3% vs 12.5% by measurement; Table S12). The 0–2 day CIF showed the same marked gradient (12.1% with full collapse vs 3.1% without, by appearance). These analyses support the robustness of the primary findings.

### Late stroke recurrences and selection bias

Details are provided in the online supplement.

## Discussion

The present pooled analysis yielded 3 principal findings. First, CNO with full collapse was associated with a substantially elevated risk of recurrent stroke within the first 2 days of the presenting event, an effect partially offset by later events in patients without full collapse. Second, in an exploratory, non-pre-specified analysis, TIA as presenting emerged as a possible additional risk factor for early recurrence, an observation that should be regarded as hypothesis-generating and that requires independent validation. Third, the combination of full collapse with TIA identified a clinical subgroup at markedly elevated early recurrence risk. The analysis incorporates individual patient data from all 3 published cohort studies designed to address this question,^7–9^ with pre-specified sensitivity analyses supporting the robustness of the primary estimates.

The 0–2 day risk window has direct clinical implications. The earlier IPDMA that informed current guidelines^5^^,^^12^ did not report preoperative recurrence rates and could not assess the effect of full collapse on this outcome. Large randomised trials (NASCET, ECST) enrolled very few cases with full collapse (*n* = 16) and assessed recurrences from randomisation rather than from the presenting event.^2^ The divergence in 90-day recurrence rates across the 3 cohorts in the present analysis is considerable and reflects substantial between-study heterogeneity. The gradient between cohorts was even steeper for ultra-early events (0–2 days). Also, there were significant between-cohort differences in baseline factors. These differences in outcomes are plausibly attributable, at least in part, to structural differences between studies. TransAtlantic Carotid Near-Occlusion Study and UCC enrolled consecutive patients using CTA-based approaches (median 2–4 days), whereas CAOS required conventional angiography with a median diagnostic delay of 12 days. This delay is particularly consequential for ultra-early events: patients with recurrent stroke within hours or days of presentation were unlikely to have been enrolled in CAOS, as recurrence preceded the angiographic diagnosis. Such survivor bias offers a plausible explanation for the 0–2 day risk in CAOS being nearly an order of magnitude lower than in TACNOS and UCC. While this heterogeneity limits the precision of pooled risk estimates and merits consideration in clinical application, it does not refute the consistent finding that full collapse markedly increases ultra-early risk within each cohort with adequate early diagnostic ascertainment. In a common population (TACNOS and UCC), the appearance- and measurement-based definitions of full collapse yielded concordant estimates; the difference in 90-day statistical significance between the full 3-cohort appearance analysis and the measurement-based analysis is therefore attributable to cohort composition—chiefly the inclusion of the lower-event CAOS cohort in the former—rather than to the definition itself.

Transient ischaemic attack as presenting event was associated with a higher recurrence risk than stroke in the overall cohort, an association not previously reported in studies of conventional ≥ 50% symptomatic stenosis,^7^^,^^18^ and which may be specific to CNO. As TIA and full collapse were independent predictors without statistically significant interaction, their combined effect is likely additive. These findings should be regarded as hypothesis-generating: TIA as presenting event was only borderline significant in the original CAOS publication^9^ and was not assessed in the TACNOS and UCC publications^7^^,^^8^; independent validation is therefore warranted. One possible biological explanation for this finding could be that, in a critically collapsed ICA, transient symptoms may reflect a haemodynamic (low flow) rather than a purely embolic mechanism, potentially heralding imminent flow failure. However, this explanation is only speculative, as the underlying mechanism associated with TIA as a presenting symptom remains unknown.

It is plausible that revascularisation might reduce the risk of stroke recurrence in symptomatic CNO, where the markedly elevated early recurrence risk observed in patients with full collapse presenting with TIA is arguably the most relevant group to consider. However, with most outcome events occurring within 48 h of presentation, several on the day of presentation, revascularisation within a few hours of presentation would be required. To date, no patient with full collapse in any identified cohort underwent CEA or CAS within 48 h with a secondary preventive goal, and data for stenting as part of thrombectomy are very limited.^3^^,^^19^ Several barriers to such ultra-early intervention exist: patients with TIA are unlikely to routinely undergo urgent CTA on arrival, the most severe variants of full collapse can be mistaken for complete occlusion,^20^ and revascularisation of CNO with full collapse is technically demanding.

Elective revascularisation series (performed beyond 48 h) consistently report higher failure rates with full collapse than without (33% vs 6% in the CAOS registry; *P* = .009), increased restenosis, and no clear reduction in 24-month stroke risk compared with medical treatment.^22^ These technical difficulties are likely compounded in the emergency setting. The single available study on emergency carotid stenting (eCAS) in CNO reported revascularisation failure rates comparable to other ICA lesions (approximately 16%), suggesting technical feasibility; however, CNO was independently associated with a significantly higher rate of intracerebral haemorrhage (42% vs 24%; OR 3.49; 95% CI, 1.63–7.48), particularly in patients with full collapse (47%), although this did not translate into higher rates of symptomatic intracerebral haemorrhage or worse 3-month functional outcomes.^19^ These observations are consistent with a haemodynamic vulnerability of full-collapse CNO, in which chronic cerebral hypoperfusion and potentially impaired autoregulation may predispose to reperfusion injury, cerebral hyperperfusion syndrome and haemorrhagic transformation after flow restoration.^21^^,^^22^ The present findings should therefore be understood as providing the risk quantification and subgroup identification required to motivate and design future trials; they do not, in themselves, support ultra-early revascularisation in routine practice outside such trials.

The relatively high 90-day recurrent stroke risk observed in patients with CNO without full collapse under the primary Kaplan–Meier analysis warrants cautious interpretation. It was partly driven by a small number of late events in a population with extensive censoring at CEA or CAS, and by a meaningful proportion of late recurrences in cases in which the stroke itself triggered the CNO diagnosis, a form of ascertainment bias that, to our knowledge, has not previously been formally evaluated in symptomatic carotid stenosis studies. The pre-specified sensitivity analysis addressing this bias yielded a substantially lower 90-day risk for CNO without full collapse (10.8% vs 17.4%). The competing risks analysis using the Aalen–Johansen estimator also lowered the estimate in this group (11.1% vs the naive 17.4%), as the high revascularisation rate generated informative censoring that the standard Kaplan–Meier method does not accommodate. Convergent results from both sensitivity analyses indicate that the apparent attenuation of the full collapse effect at 90 days under primary analysis was, in substantial part, artefactual. From a clinical management perspective, the prognosis of CNO without full collapse is considerably more favourable than that of patients with full collapse.

A significant obstacle to understanding the natural history of CNO is the lack of methodological standardisation across studies. Much of the between-study heterogeneity observed here arises from differences in diagnostic modality and timing, in the criteria used to define full collapse and in outcome ascertainment. Future studies should adopt standardised, prospective designs that take the presenting event as time zero, apply uniform CNO and full-collapse criteria, ideally with central image reading. Such standardisation is a prerequisite for the trials of urgent revascularisation that the present findings motivate.

The principal limitations of this analysis are as follows. First, between-study heterogeneity was substantial (*I*^2^ = 77.4% for 90-day risk), driven primarily by the marked difference in event rates between CAOS and the 2 Scandinavian cohorts. This heterogeneity constrains the precision and generalisability of pooled estimates; random-effects pooled rates should be interpreted in conjunction with the per-cohort figures. Second, full collapse by measurement was available only in TACNOS and UCC, limiting the scope of measurement-based analyses; the statistically significant finding for this definition (*P* = .04) reflects a selected population. Third, the high revascularisation rate within 90 days (53%) constitutes informative censoring; the competing risks analysis (Table S8) yielded cumulative incidence functions systematically lower than the primary Kaplan–Meier estimates at 90 days but preserved the relative differences between full collapse subgroups, supporting the robustness of the primary conclusions. Fourth, in patients with TIA or minor stroke, vascular imaging that is sensitive to CNO (such as CTA but not ultrasound) may be underused and this would under-ascertain CNO (included patients likely represent a more thoroughly studied—and more symptomatic—sample than the broader CNO population). This fact together with the 12-day median delay to CNO diagnosis in CAOS likely introduced substantial survivor bias, particularly for ultra-early events; this cannot be fully corrected statistically and is likely the principal driver of between-study heterogeneity. Fifth, CNO diagnosis requires advanced imaging assessments ordered on clinical grounds, introducing indication bias; included patients may represent more symptomatic cases than the broader CNO population. Sixth, no central re-reading of imaging studies was possible to perform. Seventh, the non-randomised designs of all included studies preclude any assessment of the benefit or safety of early revascularisation.

## Conclusion

Carotid near-occlusion with full collapse is associated with a markedly elevated risk of recurrent stroke within the first 2 days of the presenting event, particularly in patients presenting with TIA. These findings are consistent across pre-specified sensitivity analyses, although they must be interpreted in the context of substantial between-study heterogeneity and likely sources of bias. Prospective studies assessing the feasibility and safety of very early revascularisation are required to determine whether these early recurrences can be prevented.

## Supplementary Material

Supplementary_material_aakag100

## Data Availability

The data that support the findings of this study are not publicly available owing to privacy restrictions but are available from the corresponding author upon reasonable request. Data from each individual study are likewise available by reasonable request to the corresponding author.

## Clinical trial registration

Not applicable. This is a pooled analysis of observational cohort and registry studies and does not report a clinical trial.

## Originality and previously published material

This manuscript reports original work. It has not been published previously and is not under consideration for publication elsewhere. The individual patient data analysed here derive from 3 previously published cohort studies—TACNOS, the Umeå Carotid Cohort (UCC) and CAOS—each of which is cited in the manuscript. The present work differs from those publications in that it pools the individual patient data from all 3 cohorts and reports analyses not previously published, including the ultra-early (0–2 day) recurrent stroke risk, a formal comparison of risk by full-collapse status using all available data, and pre-specified sensitivity analyses addressing ascertainment bias and informative censoring. The dataset has not been reported in the combined form presented here.

## Permission to reproduce material from other sources

Not applicable. All figures and tables are original and were prepared by the authors; no copyrighted material from other sources is reproduced.
