# Supplementary material for: Short-term risk of recurrent stroke in symptomatic carotid near-occlusion: pooled analysis of three cohort studies
Source: Eur Stroke J. 2026 Aug 24;11(8):aakag100. doi: 10.1093/esj/aakag100 (PMC13502401; doi:10.1093/esj/aakag100)
Supplement: Supplementary file 1 [file supplementary_material_aakag100.zip › CNO_Supplemental_Online definitive.docx]

**Short-term risk of recurrent stroke in symptomatic carotid near-occlusion: pooled analysis of three cohort studies**

*Online-only supplemental material*

**Methods**

**Data harmonisation**

Definitions of some baseline factors were harmonized, resulting in slightly different definitions than in underlying publications. Data harmonisation was performed through iterative exchange and query resolution between the study authors. Variable definitions were unified according to pre-specified criteria, with discrepancies resolved by consensus. Comorbidities (hypertension, diabetes, dyslipidaemia, atrial fibrillation, ischaemic heart disease, symptomatic peripheral artery disease, and current smoking) were defined according to clinical diagnosis or treatment at the time of the presenting event. Minor differences in original variable definitions across cohorts compared with the source publications were retained when conceptually equivalent.

Missing data in key variables were handled as follows. Full collapse by appearance was available in 333 of 360 patients (93%); the 27 missing cases (from the CAOS cohort) were excluded from appearance-based analyses. Full collapse by measurement was available only in TACNOS and UCC (219 of 360, 61%), as the CAOS cohort used conventional angiography without standardised distal ICA diameter measurements. For multivariable models, complete-case analysis was used, as missing data affected fewer than 10% of patients for any individual covariate in the cohorts with complete measurement data. For patients with late recurrences, additional data were sought in individual medical records.

**Included studies**

All three eligible studies provided individual patient data for the pooled analysis. Only cases with symptomatic CNO were entered into the database. All three studies had a pre-specified aim to assess the risk of stroke recurrence in CNO, evaluated risk from the presenting event, and applied eligibility criteria that excluded cases not preliminarily eligible for revascularisation, although revascularisation itself was not required for inclusion.

Two studies comprised consecutive cases from the same tertiary centre. In both, CNO diagnosis was made by feature interpretation on CTA by two observers (central reading), and the main inclusion criterion was a 50–100% carotid stenosis. One study (TransAtlantic Carotid Near-Occlusion Study, TACNOS) was retrospective and based on review of consecutive CT angiography (CTA) scans [7]. The other (Umeå Carotid Cohort, UCC) was prospective and performed CTA systematically as part of the study [8]. Full collapse was assessed by appearance (by two observers), and a measurement-based definition of full collapse (distal ICA diameter ≤2.0 mm and/or ICA ratio ≤0.42) has been derived [4] and validated [8] in these cohorts.

One study (CAsi Oclusión Sintomática, CAOS) was a prospective multicentre CNO registry in which CNO diagnosis was based on the fulfilment of ≥2 of 4 criteria on conventional angiography by local radiologists [9]. These criteria have been described as well aligned with the feature interpretation of conventional angiography used in NASCET and ECST [2]. Full collapse was assessed by appearance only and was missing in 27 cases.

**Results**

**Late stroke recurrences and selection bias**

Twelve patients with full collapse data had a recurrent stroke more than 14 days after the presenting event. In these cases, the measurement-based definition of full collapse agreed with the appearance-based definition in 9 patients and was missing in 3; appearance-based data are therefore presented below.

In 5 of these 12 patients (3 without full collapse, 2 with full collapse), the recurrent stroke occurred 9–71 days after CNO was diagnosed, i.e. during clinical management with knowledge of the CNO diagnosis. For the remaining 7 patients (all without full collapse), the recurrent stroke itself led to the diagnostic work-up that revealed CNO: diagnosis occurred 24–91 days after the presenting event and 1–16 days after the recurrent stroke. In 5 of these 7 cases, the delay was attributable to clear mismanagement of the presenting event (failure to recognise its cerebrovascular nature, or failure to examine the carotid arteries despite a clear indication); in 2 cases the reason for the late CNO diagnosis was unclear. One of these 7 patients had an additional recurrent stroke 8 days after the first recurrence (5 days after CNO diagnosis). Case-by-case details of these 7 ascertainment-bias patients are presented in Supplemental Table 8.

In a pre-specified sensitivity analysis, the recurrent stroke that led to CNO diagnosis was treated as the presenting event in the 7 ascertainment-bias cases. Under this re-indexing, the 90-day risk of recurrent stroke was 10.8% (95% CI 5.9–15.7%) in CNO without full collapse by the appearance definition, with 3 events occurring beyond day 14, compared with 17.4% (with 10 events beyond day 14) in the primary analysis. The 90-day risk in the full collapse group was unchanged (18.7%), resulting in a statistically significant between-group difference (p=0.027; primary analysis p=0.24). With the measurement-based definition, the 90-day risk in CNO without full collapse was 13.3% (95% CI 5.5–21.1%, with 2 events beyond day 14) versus 23.7% in the primary analysis (8 events beyond day 14), and substantially lower than the 29.0% observed with full collapse (p<0.001).

**Between-study heterogeneity**

Random-effects meta-analysis using the DerSimonian–Laird estimator applied to logit-transformed Kaplan–Meier cumulative incidence estimates was used to quantify between-study heterogeneity. For the 90-day recurrent stroke risk, I² was 77.4% (Cochran’s Q=8.85, df=2, p=0.012), indicating substantial heterogeneity. For the 0–2 day risk, I² was 77.3% (Q=8.80, df=2, p=0.012). Per-cohort estimates are presented in Supplemental Table 7. This heterogeneity is largely attributable to the markedly lower event rates in CAOS, which required conventional angiography with a median diagnostic delay of 12 days, compared with the prospective CTA-based approaches used in TACNOS and UCC (median 2–4 days). Patients with very early severe recurrences were less likely to be enrolled in CAOS because their recurrence preceded the angiographic diagnosis, plausibly contributing to the divergence in observed risk between cohorts.

**Competing risks sensitivity analysis**

As 53% of patients underwent revascularisation within 90 days, an Aalen–Johansen cumulative incidence function (CIF) treating revascularisation as a competing event was computed alongside the primary Kaplan–Meier (KM) analysis. Competing-event coding was: event 1 = recurrent ischaemic stroke or retinal artery occlusion (primary outcome); event 2 = revascularisation before 90 days without prior recurrent stroke; event 0 = administrative censoring at 90 days. The Aalen–Johansen estimator was implemented with complementary log–log confidence intervals and the Marubini–Valsecchi variance formula.

Results, presented in detail in Supplemental Table 9, showed that CIF estimates were modestly lower than the corresponding KM 1−S(t) estimates, particularly in the no-full-collapse group at 90 days (–6.3 percentage points by appearance; –10.6 by measurement). This reflects the substantial fraction of these patients who underwent revascularisation before reaching day 90. Despite this reduction, the relative effect of full collapse was preserved (90-day CIF: 17.8% with full collapse vs 11.1% without, by appearance; 27.3% vs 12.5% by measurement). The marked 0–2 day gradient between full collapse and no full collapse was essentially unchanged (CIF 12.1% vs 3.1% by appearance), indicating that informative censoring by revascularisation does not materially alter the primary conclusions of the analysis.

**Supplemental figure legends**

**Supplemental Figure 1.** PRISMA flow diagram of the systematic search strategy used to identify eligible cohort studies for the pooled analysis.

**Supplemental Figure 2.** Forest plots of Kaplan–Meier estimates of the risk of recurrent stroke at 2, 14, and 90 days after the presenting event, by subgroup, including all cases. *AF, atrial fibrillation; CAOS, CAsi Oclusión Sintomática; IHD, ischaemic heart disease; PSOT, previous stroke or TIA; SPAD, symptomatic peripheral artery disease; TACNOS, TransAtlantic Carotid Near-Occlusion Study; TIA, transient ischaemic attack; UCC, Umeå Carotid Cohort.*
