# Supplementary material for: Short-term risk of recurrent stroke in symptomatic carotid near-occlusion: pooled analysis of three cohort studies
Source: Eur Stroke J. 2026 Aug 24;11(8):aakag100. doi: 10.1093/esj/aakag100 (PMC13502401; doi:10.1093/esj/aakag100)
Supplement: Supplementary file 1 [file supplementary_material_aakag100.zip › Figure-Suppl_Fig_2.pdf]

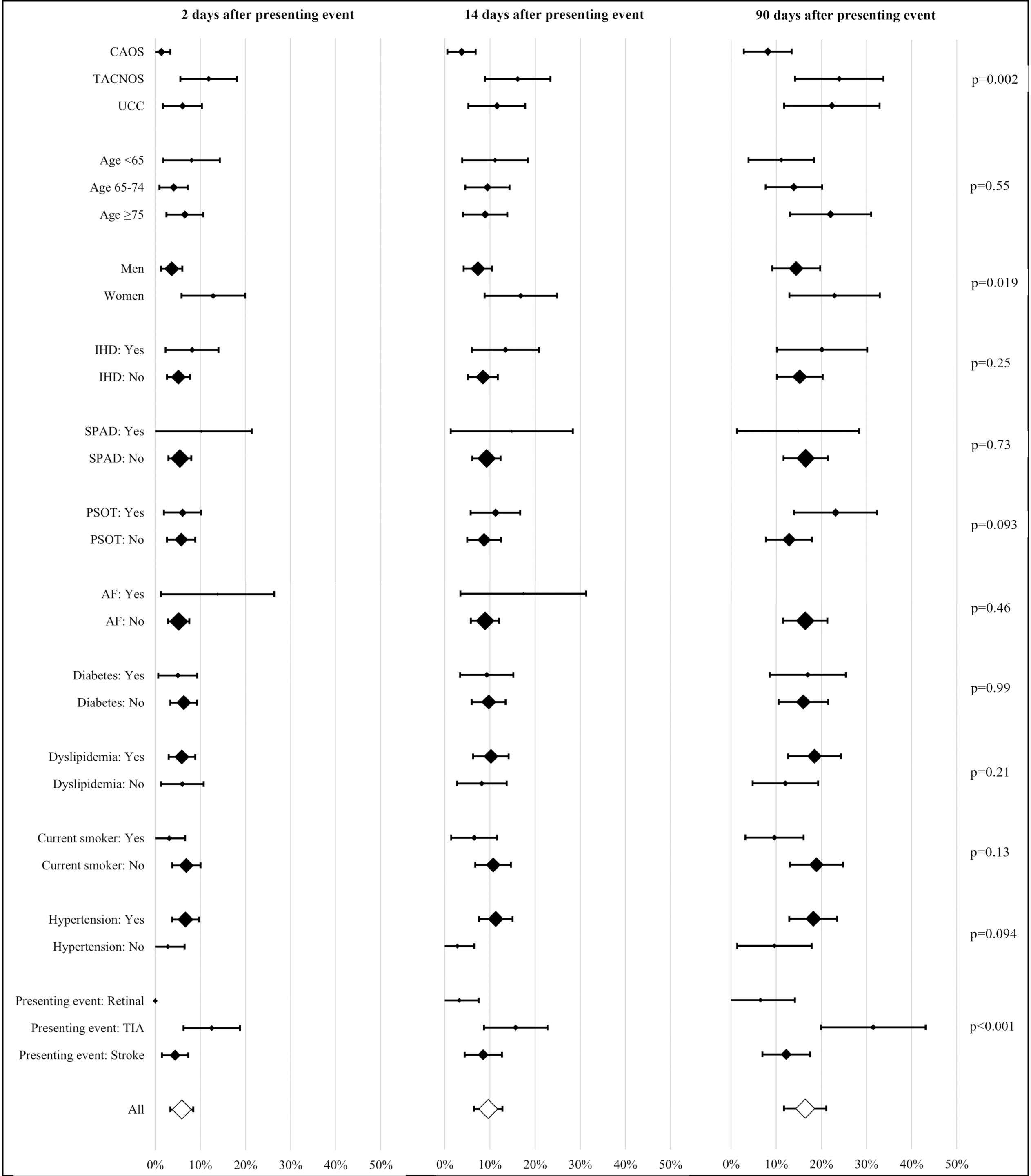

2 days after presenting event
14 days after presenting event
90 days after presenting event
CAOS
TACNOS
UCC
p=0.002
Age <65
Age 65-74
Age ≥75
p=0.55
Men
Women
p=0.019
IHD: Yes
IHD: No
p=0.25
SPAD: Yes
SPAD: No
p=0.73
PSOT: Yes
PSOT: No
p=0.093
AF: Yes
AF: No
p=0.46
Diabetes: Yes
Diabetes: No
p=0.99
Dyslipidemia: Yes
Dyslipidemia: No
p=0.21
Current smoker: Yes
Current smoker: No
p=0.13
Hypertension: Yes
Hypertension: No
p=0.094
Presenting event: Retinal
Presenting event: TIA
Presenting event: Stroke
p<0.001
All
0%
10%
20%
30%
40%
50%
