# Supplementary material for: Short-term risk of recurrent stroke in symptomatic carotid near-occlusion: pooled analysis of three cohort studies
Source: Eur Stroke J. 2026 Aug 24;11(8):aakag100. doi: 10.1093/esj/aakag100 (PMC13502401; doi:10.1093/esj/aakag100)
Supplement: Supplementary file 1 [file supplementary_material_aakag100.zip › Suppl Table 8.docx]

**Supplemental Table 8. Multivariable Cox model for recurrent stroke, adjusted for age, hypertension, diabetes and previous stroke or TIA.**

**A. Full collapse defined by appearance (n=333; 44 events at 90 days, 20 events at 0–2 days)**

| **Covariate** | **90-day adj. HR (95% CI)** | **p** | **0–2 day adj. HR (95% CI)** | **p** |
| --- | --- | --- | --- | --- |
| **Full collapse** | **1.51 (0.82–2.77)** | 0.187 | **4.10 (1.61–10.45)** | 0.003 |
| Age (per 10 years) | 1.10 (0.77–1.56) | 0.612 | 1.11 (0.66–1.87) | 0.684 |
| Hypertension | 1.87 (0.73–4.80) | 0.193 | 2.13 (0.49–9.30) | 0.313 |
| Diabetes | 0.85 (0.43–1.68) | 0.649 | 0.59 (0.19–1.80) | 0.353 |
| Previous stroke or TIA | 1.60 (0.87–2.94) | 0.128 | 1.08 (0.42–2.78) | 0.865 |

**B. Full collapse defined by measurement (n=219; 37 events at 90 days, 19 events at 0–2 days)**

| **Covariate** | **90-day adj. HR (95% CI)** | **p** | **0–2 day adj. HR (95% CI)** | **p** |
| --- | --- | --- | --- | --- |
| **Full collapse** | **2.11 (1.09–4.07)** | 0.026 | **5.19 (1.94–13.87)** | 0.001 |
| Age (per 10 years) | 1.09 (0.72–1.64) | 0.693 | 0.97 (0.54–1.73) | 0.912 |
| Hypertension | 1.88 (0.57–6.20) | 0.302 | 2.72 (0.36–20.73) | 0.334 |
| Diabetes | 0.84 (0.38–1.85) | 0.667 | 0.56 (0.16–2.00) | 0.371 |
| Previous stroke or TIA | 0.97 (0.50–1.87) | 0.924 | 0.87 (0.34–2.24) | 0.777 |

*Adjusted hazard ratios (95% CI) from a single Cox model entering full collapse and the four specified comorbidities (age, hypertension, diabetes, and previous stroke or TIA — at any time before the presenting event) simultaneously. None of the four comorbidities was associated with recurrence in any model. HR, hazard ratio; CI, confidence interval; TIA, transient ischaemic attack.*

*The full-collapse effect was essentially unchanged from the primary parsimonious models (Tables 2–3), indicating that these comorbidities did not confound it.*

*Robustness to structural adjustment: when cohort and TIA as presenting event were additionally included, the full-collapse HR was 1.59 (0.82–3.07) for appearance at 90 days, 5.08 (1.84–14.00) for appearance at 0–2 days, 2.00 (1.00–3.99) for measurement at 90 days, and 5.17 (1.89–14.09) for measurement at 0–2 days.*

*Antithrombotic therapy and recent (crescendo) events preceding the presenting event could not be included, as they were not captured uniformly across the three cohorts in the harmonised database (see responses to major comments 4 and 5).*

*With 44 (90-day) and 20 (0–2 day) recurrent events, the events-per-variable ratio is low; this model is exploratory and hypothesis-generating, and the confidence intervals are correspondingly wide.*
