# Supplementary material for: Short-term risk of recurrent stroke in symptomatic carotid near-occlusion: pooled analysis of three cohort studies
Source: Eur Stroke J. 2026 Aug 24;11(8):aakag100. doi: 10.1093/esj/aakag100 (PMC13502401; doi:10.1093/esj/aakag100)
Supplement: Supplementary file 1 [file supplementary_material_aakag100.zip › Supplemental Table 1.docx]

**Supplemental Table 1. Peri-procedural stroke or death within 30 days of revascularisation, by full collapse status and procedure type.**

**A. Full collapse defined by appearance (revascularised patients, n=191)**

| **Group** | **CEA** | **CAS** | **All revascularised** | **p †** |
| --- | --- | --- | --- | --- |
| CNO without full collapse | 2/117 (1.7) | 2/31 (6.5) | 4/148 (2.7) | Ref |
| CNO with full collapse | 4/16 (25.0) | 1/13 (7.7) | 5/29 (17.2) | 0.007 |
| Full collapse status unknown ‡ | 0/1 | 0/13 | 0/14 | – |
| **All patients** | 6/134 (4.5) | 3/57 (5.3) | **9/191 (4.7)** | – |

**B. Full collapse defined by measurement (revascularised patients, TACNOS and UCC, n=132)**

| **Group** | **CEA** | **CAS** | **All revascularised** | **p †** |
| --- | --- | --- | --- | --- |
| CNO without full collapse | 2/103 (1.9) | 1/11 (9.1) | 3/114 (2.6) | Ref |
| CNO with full collapse | 1/12 (8.3) | 1/6 (16.7) | 2/18 (11.1) | 0.137 |
| **All patients** | 3/115 (2.6) | 2/17 (11.8) | **5/132 (3.8)** | – |
