# Supplementary material for: Short-term risk of recurrent stroke in symptomatic carotid near-occlusion: pooled analysis of three cohort studies
Source: Eur Stroke J. 2026 Aug 24;11(8):aakag100. doi: 10.1093/esj/aakag100 (PMC13502401; doi:10.1093/esj/aakag100)
Supplement: Supplementary file 1 [file supplementary_material_aakag100.zip › Supplemental Table 5.docx]

**Supplemental Table 5. Kaplan–Meier risk of recurrent stroke and Cox hazard ratios for full collapse, by definition, in the common TACNOS+UCC population (n=219).**

| **Definition** | **Group** | **0–2 d, % (95% CI)** | **90 d, % (95% CI)** | **Adj. HR 0–2 d** | **Adj. HR 90 d** |
| --- | --- | --- | --- | --- | --- |
| Appearance | With FC (n=66) | 18.6 (9.1–28.0) | 27.7 (16.4–39.1) | 4.9 (1.9–12.7) | 1.8 (0.9–3.5) |
| Appearance | Without FC (n=153) | 4.6 (1.3–7.9) | 23.1 (12.5–33.7) | ref | ref |
| Measurement | With FC (n=66) | 20.1 (10.3–29.8) | 29.0 (17.6–40.4) | 4.8 (1.8–12.8) | 2.0 (1.0–3.9) |
| Measurement | Without FC (n=153) | 4.0 (0.9–7.1) | 23.1 (12.5–33.8) | ref | ref |

*FC, full collapse; CI, confidence interval; HR, hazard ratio. Recurrent stroke = ipsilateral ischaemic stroke or retinal artery occlusion. Kaplan–Meier risk with Greenwood 95% CI. Adjusted HRs are for full collapse, from Cox models built with the same covariate screening (p<0.10) as the main analysis; the 0–2 day appearance model adjusted for female sex and TIA as presenting event, the 90-day appearance model for retinal and TIA presenting event. Log-rank (appearance, restricted): 0–2 days p<0.001; 90 days p=0.084. Agreement between definitions where both were available: 94.5%.*
