# Supplementary material for: Short-term risk of recurrent stroke in symptomatic carotid near-occlusion: pooled analysis of three cohort studies
Source: Eur Stroke J. 2026 Aug 24;11(8):aakag100. doi: 10.1093/esj/aakag100 (PMC13502401; doi:10.1093/esj/aakag100)
Supplement: Supplementary file 1 [file supplementary_material_aakag100.zip › Supplemental_Table_10.docx]

**Supplemental Table 10. Post-hoc analyses for risk of recurrent stroke after presenting event in those with and without TIA as presenting event combined with full collapse status.**

|  | **Risk** |  | **Risk** | **p (log rank)** |
| --- | --- | --- | --- | --- |
| **2-day risk, FC defined by appearance, n=333** | | | | |
| TIA positive & FC positive | 33.3 | TIA negative, FC negative | 2.7 | <.001 |
|  |  | TIA negative, FC positive | 5.1 | <.001 |
|  |  | TIA positive, FC negative | 4.2 | <.001 |
| TIA negative & FC positive | 5.1 | TIA positive, FC negative | 4.2 | .79 |
|  |  | TIA negative, FC negative | 2.7 | .34 |
| TIA positive & FC negative | 4.2 | TIA negative, FC negative | 2.7 | .54 |
| **2-day risk, FC defined by measurement, n=219** | | | | |
| TIA positive & FC positive | 42.1 | TIA negative, FC negative | 3.0 | <.001 |
|  |  | TIA negative, FC positive | 10.9 | <.001 |
|  |  | TIA positive, FC negative | 5.9 | <.001 |
| TIA negative & FC positive | 10.9 | TIA positive, FC negative | 5.9 | .37 |
|  |  | TIA negative, FC negative | 3.0 | .045 |
| TIA positive & FC negative | 5.9 | TIA negative, FC negative | 3.0 | .38 |
| **90-day risk, FC defined by appearance, n=333** | | | | |
| TIA positive & FC positive | 37.8 | TIA negative, FC negative | 12.2 | <.001 |
|  |  | TIA negative, FC positive | 12.3 | .001 |
|  |  | TIA positive, FC negative | 29.8 | .066 |
| TIA negative & FC positive | 12.3 | TIA positive, FC negative | 29.8 | .12 |
|  |  | TIA negative, FC negative | 12.2 | .73 |
| TIA positive & FC negative | 29.8 | TIA negative, FC negative | 12.2 | .041 |
| **90-day risk, FC defined by measurement, n=219** | | | | |
| TIA positive & FC positive | 50.4 | TIA negative, FC negative | 17.8 | <.001 |
|  |  | TIA negative, FC positive | 20.5 | .014 |
|  |  | TIA positive, FC negative | 33.1 | .011 |
| TIA negative & FC positive | 20.5 | TIA positive, FC negative | 33.1 | .90 |
|  |  | TIA negative, FC negative | 17.8 | .20 |
| TIA positive & FC negative | 33.1 | TIA negative, FC negative | 17.8 | .14 |

*FC: Full collapse. TIA: Transient ischemic attack. Four 4-group assessments (both definitions of full collapse and 2-day and 90-day) all had p<.001.*
