# Supplementary material for: Short-term risk of recurrent stroke in symptomatic carotid near-occlusion: pooled analysis of three cohort studies
Source: Eur Stroke J. 2026 Aug 24;11(8):aakag100. doi: 10.1093/esj/aakag100 (PMC13502401; doi:10.1093/esj/aakag100)
Supplement: Supplementary file 1 [file supplementary_material_aakag100.zip › Supplemental_Table_11.docx]

**Supplemental Table 11. Case-by-case characteristics of patients with ascertainment bias (recurrent stroke preceding CNO diagnosis).**

| **Case** | **Cohort** | **Presenting event** | **Days from PE to recurrence** | **Days from PE to CNO diagnosis** | **Days from recurrence to diagnosis** | **Full collapse status** |
| --- | --- | --- | --- | --- | --- | --- |
| 1 | UCC | Retinal | 25 | 27 | 2 | Without full collapse |
| 2 | TACNOS | TIA | 30 | 33 | 3 | Without full collapse |
| 3 | TACNOS | TIA | 62 | 65 | 3 | Without full collapse |
| 4 | UCC | TIA | 20 | 24 | 4 | Without full collapse |
| 5 | UCC | Stroke | 21 | 28 | 7 | Without full collapse |
| 6 | UCC | Stroke | 42 | 58 | 16 | Without full collapse |
| 7 | CAOS | Stroke | 90 | 91 | 1 | Without full collapse |

***Summary statistics for the 7 ascertainment bias cases:***

- *Days from PE to recurrence: range 20–90, median 30*
- *Days from PE to CNO diagnosis: range 24–91, median 33*
- *Days from recurrence to diagnosis: range 1–16, median 3*
- *Distribution by cohort: TACNOS 2, UCC 4, CAOS 1; all 7 cases without full collapse*

*CNO, carotid near-occlusion; PE, presenting event; TIA, transient ischaemic attack; TACNOS, TransAtlantic Carotid Near-Occlusion Study; UCC, Umeå Carotid Cohort; CAOS, CAsi Oclusión Sintomática. These cases represent a form of ascertainment bias in which recurrent stroke prompted the imaging assessment that ultimately led to CNO diagnosis. Re-indexing these events to the time of recurrent stroke (treating recurrence as the presenting event) yielded a 90-day recurrence risk of 10.8% in CNO without full collapse, compared with 17.4% in the primary analysis, and resulted in a statistically significant between-group difference with full collapse (p=0.027).*
