# Supplementary material for: Short-term risk of recurrent stroke in symptomatic carotid near-occlusion: pooled analysis of three cohort studies
Source: Eur Stroke J. 2026 Aug 24;11(8):aakag100. doi: 10.1093/esj/aakag100 (PMC13502401; doi:10.1093/esj/aakag100)
Supplement: Supplementary file 1 [file supplementary_material_aakag100.zip › Supplemental_Table_12.docx]

**Supplemental Table 12. Competing risks sensitivity analysis: Aalen–Johansen cumulative incidence function (CIF) treating revascularisation as a competing event, compared with the primary Kaplan–Meier (KM) estimates.**

***A. 90-day estimates***

| **Subgroup** | **N** | **Events** | **KM 1–S (%) (95% CI)** | **CIF (%)** | **CIF 95% CI (%)** | **Difference** |
| --- | --- | --- | --- | --- | --- | --- |
| **Visual definition (n=333)** | | | | | | |
| Overall | 333 | 44 | 16.9 (12.0–21.8) | 13.3 | 9.8–17.7 | –3.6 |
| CNO with full collapse | 107 | 19 | 18.7 (11.1–26.3) | 17.8 | 11.3–27.4 | –0.9 |
| CNO without full collapse | 226 | 25 | 17.4 (10.5–24.3) | 11.1 | 7.5–16.3 | –6.3 |
| **Measurement definition (n=219)** | | | | | | |
| CNO with full collapse | 66 | 18 | 29.0 (17.6–40.4) | 27.3 | 17.1–41.9 | –1.7 |
| CNO without full collapse | 153 | 19 | 23.1 (12.5–33.7) | 12.5 | 7.9–19.4 | –10.6 |

***B. 0–2 day estimates***

| **Subgroup** | **N** | **Events** | **KM 1–S (%) (95% CI)** | **CIF (%)** | **CIF 95% CI (%)** | **Difference** |
| --- | --- | --- | --- | --- | --- | --- |
| **Visual definition (n=333)** | | | | | | |
| CNO with full collapse | 107 | 13 | 12.3 (6.0–18.3) | 12.1 | 7.1–20.4 | –0.2 |
| CNO without full collapse | 226 | 7 | 3.1 (0.7–5.5) | 3.1 | 1.5–6.4 | 0.0 |

*Competing event coding: of 333 patients (visual definition), 44 had a recurrent stroke (event of interest), 161 underwent revascularisation without prior recurrent stroke (competing event), and 128 reached day 90 event-free (administrative censoring). CIF, cumulative incidence function (Aalen–Johansen estimator); CI, confidence interval; KM, Kaplan–Meier. As expected when competing events are present, the CIF estimates are modestly lower than the corresponding KM 1–S estimates, particularly in the no full collapse group at 90 days (–6.3 percentage points by appearance; –10.6 by measurement), reflecting the substantial fraction of patients without full collapse who underwent revascularisation before 90 days. The relative effect of full collapse on the cumulative incidence is preserved, and the marked 0–2 day gradient between full collapse and no full collapse remains essentially unchanged. These results indicate that informative censoring by revascularisation does not materially alter the primary conclusions of the analysis.*
