# Supplementary material for: Short-term risk of recurrent stroke in symptomatic carotid near-occlusion: pooled analysis of three cohort studies
Source: Eur Stroke J. 2026 Aug 24;11(8):aakag100. doi: 10.1093/esj/aakag100 (PMC13502401; doi:10.1093/esj/aakag100)
Supplement: Supplementary file 1 [file supplementary_material_aakag100.zip › Supplemental_Table_2.docx]

**Supplemental Table 2. Per-cohort Kaplan–Meier event rates and random-effects meta-analytic heterogeneity statistics.**

***A. 90-day recurrent stroke risk (all patients, n=360)***

| **Cohort** | **N** | **Events** | **KM risk (%)** | **95% CI (%)** | **I² (%)** | **Q** | **p** |
| --- | --- | --- | --- | --- | --- | --- | --- |
| TACNOS | 101 | 20 | 23.9 | 15.6 – 35.5 |  |  |  |
| UCC | 118 | 17 | 22.3 | 13.7 – 35.1 |  |  |  |
| CAOS | 141 | 9 | 8.1 | 4.2 – 15.2 |  |  |  |
| **RE pooled estimate** | **360** | **46** | **17.0** | **8.8 – 30.2** | **77.4** | **8.85** | **0.012** |

***B. 0–2 day recurrent stroke risk (all patients, n=360)***

| **Cohort** | **N** | **Events** | **KM risk (%)** | **95% CI (%)** | **I² (%)** | **Q** | **p** |
| --- | --- | --- | --- | --- | --- | --- | --- |
| TACNOS | 101 | 12 | 11.9 | 6.9 – 20.0 |  |  |  |
| UCC | 118 | 7 | 6.1 | 2.9 – 12.3 |  |  |  |
| CAOS | 141 | 2 | 1.4 | 0.4 – 5.6 |  |  |  |
| **RE pooled estimate** | **360** | **21** | **5.6** | **2.0 – 14.5** | **77.3** | **8.80** | **0.012** |

***C. 90-day recurrent stroke risk by full collapse status – visual definition (n=333)***

| **Group / Cohort** | **N** | **Events** | **KM risk (%)** | **95% CI (%)** | **I² (%)** | **Q** | **p** |
| --- | --- | --- | --- | --- | --- | --- | --- |
| **Full collapse (n=107)** |  |  |  |  | **69.8** | **6.63** | **0.036** |
| TACNOS | 43 | 12 | 29.1 | 17.6 – 45.8 |  |  |  |
| UCC | 23 | 5 | 24.1 | 10.7 – 49.1 |  |  |  |
| CAOS | 41 | 2 | 4.9 | 1.2 – 18.1 |  |  |  |
| **RE pooled** | **107** | **19** | **18.0** | **7.0 – 39.1** |  |  |  |
| **No full collapse (n=226)** |  |  |  |  | **37.6** | **3.20** | **0.202** |
| TACNOS | 58 | 8 | 21.9 | 10.1 – 43.6 |  |  |  |
| UCC | 95 | 12 | 23.6 | 12.9 – 40.7 |  |  |  |
| CAOS | 73 | 5 | 9.6 | 4.0 – 22.1 |  |  |  |
| **RE pooled** | **226** | **25** | **17.8** | **10.2 – 29.3** |  |  |  |

*KM, Kaplan–Meier; CI, confidence interval; RE, random effects; I², between-study heterogeneity statistic; Q, Cochran's Q; TACNOS, TransAtlantic Carotid Near-Occlusion Study; UCC, Umeå Carotid Cohort; CAOS, CAsi Oclusión Sintomática. Heterogeneity was calculated using the DerSimonian–Laird random-effects estimator applied to logit-transformed Kaplan–Meier cumulative incidence estimates. Random-effects pooled estimates are back-transformed to the probability scale; 95% confidence intervals for pooled estimates are based on the random-effects standard error. Full collapse subgroup analyses by measurement definition (n=219) are presented in Table 2 and Supplemental Table 2.*
