# Supplementary material for: Short-term risk of recurrent stroke in symptomatic carotid near-occlusion: pooled analysis of three cohort studies
Source: Eur Stroke J. 2026 Aug 24;11(8):aakag100. doi: 10.1093/esj/aakag100 (PMC13502401; doi:10.1093/esj/aakag100)
Supplement: Supplementary file 1 [file supplementary_material_aakag100.zip › Supplemental_Table_3.docx]

**Supplemental Table 3. Kaplan–Meier assessment of recurrent stroke (i.e. recurrent ipsilateral ischemic stroke or retinal artery occlusion).**

|  | **All CNO^a^** | | **CNO without full collapse** | | **CNO with full collapse** | |
| --- | --- | --- | --- | --- | --- | --- |
|  | **Cumulative events** | **% risk (95%CI)** | **Cumulative events** | **% risk (95%CI)** | **Cumulative events** | **% risk (95%CI)** |
| **Full collapse defined by appearance, n=333^b^** | | | | | | |
| Day 0 | 9 | 2.7 (0.9 – 4.4) | 3 | 1.3 (-0.3 – 2.9) | 6 | 5.6 (1.3 – 9.9) |
| Day 2 | 20 | 6.1 (3.5 – 8.6) | 7 | 3.1 (0.7 – 5.5)^c^ | 13 | 12.3 (6 – 18.6)^c^ |
| Day 7 | 30 | 9.3 (6.2 – 12.4) | 15 | 7.1 (3.6 – 10.6) | 15 | 14.2 (7.6 – 20.8) |
| Day 14 | 32 | 10.1 (6.8 – 13.4) | 15 | 7.1 (3.6 – 10.6) | 17 | 16.3 (9.3 – 23.3) |
| Day 30 | 38 | 13.2 (9.1 – 17.3) | 20 | 11.6 (6.5 – 16.7) | 18 | 17.4 (10.3 – 24.5) |
| Day 90 | 44 | 16.9 (12.0 – 21.8) | 25 | 17.4 (10.5 – 24.3)^c^ | 19 | 18.7 (11.1 – 26.3)^c^ |
| **Full collapse defined by measurement, n=219^b^** | | | | | | |
| Day 0 | 9 | 4.1 (1.5 – 6.7) | 2 | 1.3 (-0.5 – 3.1) | 7 | 10.6 (3.1 – 18.1) |
| Day 2 | 19 | 8.8 (5.1 – 12.5) | 6 | 4.0 (0.9 – 7.1)^d^ | 13 | 20.1 (10.3 – 29.9)^d^ |
| Day 7 | 26 | 12.3 (7.8 – 16.8) | 11 | 7.6 (3.3 – 11.9) | 15 | 23.3 (12.9 – 33.7) |
| Day 14 | 28 | 13.6 (8.9 – 18.3) | 11 | 7.6 (3.3 – 11.9) | 17 | 26.9 (15.9 – 37.9) |
| Day 30 | 33 | 18.2 (12.1 – 24.3) | 15 | 14.2 (6.8 – 21.6) | 18 | 29.0 (17.6 – 40.4) |
| Day 90 | 37 | 22.9 (15.6 – 30.2) | 19 | 23.1 (12.5 – 33.7)^d^ | 18 | 29.0 (17.6 – 40.4)^d^ |

*CAOS: CAsi Oclusión Síntomatica. CI: Confidence interval. CNO: Carotid near-occlusion. TACNOS: TransAtlantic Carotid Near-Occlusion Study. UCC: Umeå Carotid Cohort. a All 333 in appearance analysis and all 219 in measurement analysis. b Full collapse by appearance available for all but 27 cases in CAOS. Full collapse by measurement only available in TACNOS and UCC study. c p-values for log rank were: 0–90 day p=.24, 0–2 days p=.001, 3–90 days p=.19. d p-values for log rank were: 0–90 day p=.020, 0–2 days p<.001, 3–90 days p=.57.*
