# Supplementary material for: Short-term risk of recurrent stroke in symptomatic carotid near-occlusion: pooled analysis of three cohort studies
Source: Eur Stroke J. 2026 Aug 24;11(8):aakag100. doi: 10.1093/esj/aakag100 (PMC13502401; doi:10.1093/esj/aakag100)
Supplement: Supplementary file 1 [file supplementary_material_aakag100.zip › Supplemental_Table_4.docx]

**Supplemental Table 4. Hazard ratio (HR) for recurrent stroke within 90 days of presenting event. Co-variates not included in either multivariable model (due to p>0.1 in the bivariable analysis).**

|  | **Bivariable HR (95%CI)** | **p** | **Model 1** | | **Model 2** | |
| --- | --- | --- | --- | --- | --- | --- |
|  |  |  | **Multivariable HR (95%CI)** | **p** | **Multivariable HR (95%CI)** | **p** |
| **Full collapse defined by appearance, n=333** | | | | | | |
| Age | 1.0 (1.0-1.0) | .53 | Not used | - | Not used | - |
| Age (10 year increase) | 1.1 (0.8 – 1.6) | .53 | Not used | - | Not used | - |
| Ischemic heart disease | 1.2 (0.6-2.4) | .54 | Not used | - | Not used | - |
| Symptomatic peripheral artery disease | 1.0 (0.3-3.3) | .99 | Not used | - | Not used | - |
| **Any stroke/TIA >6 months before PE** | 1.0 (0.5–2.0) | .98 | Not used | - | Not used | - |
| Atrial fibrillation | 1.4 (0.6-3.6) | .45 | Not used | - | Not used | - |
| Diabetes | 0.9 (0.5-1.7) | .75 | Not used | - | Not used | - |
| Dyslipidemia | 1.5 (0.7-3.2) | .25 | Not used | - | Not used | - |
| Current smoker | 0.6 (0.3-1.3) | .17 | Not used | - | Not used | - |
| Hypertension | 2.0 (0.8-5.0) | .15 | Not used | - | Not used | - |
| **Recent ipsilateral ischaemic event ≤7 d before PE** | 1.7 (0.9–3.4) | .117 | Not used | - | Not used | - |
| **Full collapse defined by measurement, n=219** | | | | | | |
| Cohort: TACNOS^a^ | 1.3 (0.7-2.4) | .46 | Not used | - | Not used | - |
| Age | 1.0 (1.0-1.0) | .90 | Not used | - | Not used | - |
| Age (10 year increase) | 1.0 (0.7-1.5) | .90 | Not used | - | Not used | - |
| Women | 1.5 (0.8-2.8) | .26 | Not used | - | Not used | - |
| Ischemic heart disease | 1.0 (0.5-2.1) | .95 | Not used | - | Not used | - |
| Symptomatic peripheral artery disease | 1.2 (0.4-3.9) | .76 | Not used | - | Not used | - |
| **Any stroke/TIA >6 months before PE** | 0.6 (0.2–1.4) | .24 | Not used | - | Not used | - |
| Atrial fibrillation | 1.1 (0.4-3.1) | .88 | Not used | - | Not used | - |
| Diabetes | 1.0 (0.4-2.0) | .89 | Not used | - | Not used | - |
| Dyslipidemia | 1.0 (0.4-2.4) | .98 | Not used | - | Not used | - |
| Current smoker | 0.7 (0.3-1.6) | .40 | Not used | - | Not used | - |
| Hypertension | 2.0 (0.6-6.4) | .27 | Not used | - | Not used | - |
| **Recent ipsilateral ischaemic event ≤7 d before PE** | 1.0 (0.4–2.2) | .958 | Not used | - | Not used | - |

*CAOS: CAsi Oclusión Síntomatica. HR: Hazard ratio. TACNOS: Transatlantic carotid near-occlusion study. TIA: Transient ischemic attack. UCC: Umeå Carotid Cohort. Two separate analyses, one for the population of 333 cases with data on full collapse by visual assessment, one for the population of 219 cases with full collapse by measurement assessment. Co-variates with a possible association (p<0.10) with outcome in each of the two populations were included in the multivariable models. Model 1: Standard approach. Model 2: Merging presenting event and full collapse and controlling for remaining co-variates. a UCC as reference, no data available in CAOS.*
