# Supplementary material for: Short-term risk of recurrent stroke in symptomatic carotid near-occlusion: pooled analysis of three cohort studies
Source: Eur Stroke J. 2026 Aug 24;11(8):aakag100. doi: 10.1093/esj/aakag100 (PMC13502401; doi:10.1093/esj/aakag100)
Supplement: Supplementary file 1 [file supplementary_material_aakag100.zip › Supplemental_Table_6.docx]

**Supplemental Table 6. Hazard ratio (HR) for recurrent stroke at 0–2 days of presenting event. Co-variates not included in either multivariable model (due to p>0.1 in the bivariable analysis).**

|  | **Bivariable HR (95%CI)** | **p** | **Model 1** | | **Model 2** | |
| --- | --- | --- | --- | --- | --- | --- |
|  |  |  | **Multivariable HR (95%CI)** | **p** | **Multivariable HR (95%CI)** | **p** |
| **Full collapse defined by appearance, n=333** | | | | | | |
| Age | 1.0 (1.0 – 1.1) | .90 | Not used | - | Not used | - |
| Age (10 year increase) | 1.0 (0.6 – 1.7) | .90 | Not used | - | Not used | - |
| Ischemic heart disease | 1.4 (0.5 – 3.5) | .53 | Not used | - | Not used | - |
| Symptomatic peripheral artery disease | 1.4 (0.3 – 6.1) | .64 | Not used | - | Not used | - |
| **Any stroke/TIA >6 months before PE** | 0.6 (0.2–2.1) | .43 | Not used | - | Not used | - |
| Diabetes | 0.6 (0.2 – 1.9) | .44 | Not used | - | Not used | - |
| Dyslipidemia | 0.9 (0.3 – 2.3) | .77 | Not used | - | Not used | - |
| Current smoker | 0.5 (0.1 – 1.7) | .26 | Not used | - | Not used | - |
| Hypertension | 2.3 (0.5 – 9.8) | .27 | Not used | - | Not used | - |
| **Recent ipsilateral ischaemic event ≤7 d before PE** | 1.6 (0.6–4.3) | .393 | Not used | - | Not used | - |
| **Full collapse defined by measurement, n=219** | | | | | | |
| Cohort: TACNOS^a^ | 2.0 (0.8 – 5.1) | .14 | Not used | - | Not used | - |
| Age | 1.0 (0.9 – 1.0) | .52 | Not used | - | Not used | - |
| Age (10 year increase) | 0.8 (0.5 – 1.4) | .52 | Not used | - | Not used | - |
| Ischemic heart disease | 1.1 (0.4 – 2.9) | .82 | Not used | - | Not used | - |
| Symptomatic peripheral artery disease | 1.5 (0.3 – 6.4) | .60 | Not used | - | Not used | - |
| **Any stroke/TIA >6 months before PE** | 0.6 (0.2–2.0) | .39 | Not used | - | Not used | - |
| Atrial fibrillation | 1.7 (0.5 – 5.8) | .40 | Not used | - | Not used | - |
| Diabetes | 0.6 (0.2 – 2.1) | .42 | Not used | - | Not used | - |
| Dyslipidemia | 0.5 (0.2 – 1.5) | .21 | Not used | - | Not used | - |
| Current smoker | 0.7 (0.2 – 2.4) | .58 | Not used | - | Not used | - |
| Hypertension | 3.3 (0.4 – 24.5) | .25 | Not used | - | Not used | - |
| **Recent ipsilateral ischaemic event ≤7 d before PE** | 1.1 (0.4–3.3) | .877 | Not used | - | Not used | - |

*CAOS: CAsi Oclusión Síntomatica. HR: Hazard ratio. TACNOS: Transatlantic carotid near-occlusion study. TIA: Transient ischemic attack. UCC: Umeå Carotid Cohort. Same analysis approach and models as in Table 3. a UCC as reference, no data available in CAOS.*
