# Supplementary material for: Short-term risk of recurrent stroke in symptomatic carotid near-occlusion: pooled analysis of three cohort studies
Source: Eur Stroke J. 2026 Aug 24;11(8):aakag100. doi: 10.1093/esj/aakag100 (PMC13502401; doi:10.1093/esj/aakag100)
Supplement: Supplementary file 1 [file supplementary_material_aakag100.zip › Supplemental_Table_7.docx]

**Supplemental Table 7. Multivariable analysis. Hazard ratio (HR) for recurrent stroke 3–90 days of presenting event.**

|  | **Bivariable HR (95%CI)** | **p** | **Model 1** | | **Model 2** | |
| --- | --- | --- | --- | --- | --- | --- |
|  |  |  | **Multivariable HR (95%CI)** | **p** | **Multivariable HR (95%CI)** | **p** |
| **Full collapse defined by appearance, n=302** | | | | | | |
| Full collapse | 0.5 (0.2 – 1.4) | .20 | 0.6 (0.2 – 1.6) | .34 | Not used | - |
| Cohort: CAOS | Ref | - | Ref | - | Ref | - |
| Cohort: TACNOS | 2.1 (0.7 – 6.0) | .18 | 1.6 (0.5-4.9) | .38 | 1.5 (0.5-4.6) | .45 |
| Cohort: UCC | 2.6 (0.9 – 7.2) | .069 | 1.5 (0.5 – 4.6) | .46 | 1.5 (0.5 – 4.5) | .50 |
| Age | 1.0 (1.0 – 1.1) | .47 | Not used | - | Not used | - |
| Age (10-year increase) | 1.2 (0.7 – 1.9) | .47 | Not used | - | Not used | - |
| Women | 1.1 (0.4 – 2.6) | .92 | Not used | - | Not used | - |
| Ischemic heart disease | 1.1 (0.4 – 2.8) | .80 | Not used | - | Not used | - |
| Symptomatic peripheral artery disease | 0.6 (0.1 – 4.8) | .67 | Not used | - | Not used | - |
| **Any stroke/TIA >6 months before PE** | 1.4 (0.6–3.4) | .45 | Not used | - | Not used | - |
| Atrial fibrillation | 0.5 (0.1 – 3.6) | .49 | Not used | - | Not used | - |
| Diabetes | 1.1 (0.5 – 2.6) | .79 | Not used | - | Not used | - |
| Dyslipidemia | 2.9 (0.9 – 9.8) | .083 | 2.1 (0.6 – 7.6) | .26 | 2.3 (0.6 – 8.2) | .22 |
| Current smoker | 0.7 (0.2 – 1.8) | .42 | Not used | - | Not used | - |
| Hypertension | 1.8 (0.5 – 6.0) | .34 | Not used | - | Not used | - |
| **Recent ipsilateral ischaemic event ≤7 d before PE** | 1.9 (0.8–4.8) | .176 | Not used | - | Not used | - |
| Presenting event: Stroke | Ref | - | Ref | - | Ref | - |
| Presenting event: Retinal | 0.8 (0.2 – 2.8) | .72 | Not used | - | Not used | - |
| Presenting event: TIA^a^ | 2.0 (0.8 – 4.7) | .12 | Not used | - | Not used | - |
| Full collapse and presenting event TIA | 0.5 (0.1 – 4.0) | .54 | Not used | - | 0.5 (0.1 – 3.9) | .52 |
| **Full collapse defined by measurement, n=193** | | | | | | |
| Full collapse | 0.7 (0.3 – 2.1) | .74 | 0.7 (0.3 – 2.1) | .74 | Not used | - |
| Cohort: TACNOS^b^ | 0.8 (0.3 – 2.0) | .64 | Not used | - | Not used | - |
| Age | 1.0 (1.0 – 1.1) | .42 | Not used | - | Not used | - |
| Age (10 year increase) | 1.3 (0.7 – 2.2) | .42 | Not used | - | Not used | - |
| Women | 0.7 (0.2 – 2.0) | .46 | Not used | - | Not used | - |
| Ischemic heart disease | 0.9 (0.3 – 2.6) | .89 | Not used | - | Not used | - |
| Symptomatic peripheral artery disease | 0.9 (0.1 – 6.6) | .90 | Not used | - | Not used | - |
| **Any stroke/TIA >6 months before PE** | 0.6 (0.2–2.1) | .41 | Not used | - | Not used | - |
| Atrial fibrillation | 0.5 (0.1 – 3.9) | .52 | Not used | - | Not used | - |
| Diabetes | 1.4 (0.5 – 3.6) | .54 | Not used | - | Not used | - |
| Dyslipidemia | 3.5 (0.5 – 26.4) | .22 | Not used | - | Not used | - |
| Current smoker | 0.7 (0.2 – 2.3) | .53 | Not used | - | Not used | - |
| Hypertension | 1.3 (0.3 – 5.7) | .73 | Not used | - | Not used | - |
| **Recent ipsilateral ischaemic event ≤7 d before PE** | 0.9 (0.2–3.0) | .812 | Not used | - | Not used | - |
| Presenting event: Stroke | Ref | - | Ref | - | Ref | - |
| Presenting event: Retinal | 0.4 (0.1 – 1.6) | .18 | Not used | - | Not used | - |
| Presenting event: TIA^c^ | 1.3 (0.5 – 3.5) | .60 | Not used | - | Not used | - |
| Full collapse and presenting event TIA | 0.9 (0.1 – 6.6) | .90 | Not used | - | 0.9 (0.1 – 6.6) | .90 |

*CAOS: CAsi Oclusión Síntomatica. HR: Hazard ratio. TACNOS: Transatlantic carotid near-occlusion study. TIA: Transient ischemic attack. UCC: Umeå Carotid Cohort. Same analysis approach and models as in Table 3 but day 3 is changed to day 0 (index) and 31 patients from appearance-based assessment and 26 patients from the measurement-based assessments were excluded due to event or censoring during day 0–2. a When using non-TIA events as reference, HR 2.1 (0.9 – 4.7), p=0.073 in bivariable and 1.7 (0.8 – 4.0), p=0.20 in model 1. b UCC as reference, no data available in CAOS. c When using non-TIA events as reference, HR 1.7 (0.7 – 4.5), p=0.25 in bivariable and 1.7 (0.6 – 4.4), p=0.29 in model 1.*
