# Supplementary material for: Short-term risk of recurrent stroke in symptomatic carotid near-occlusion: pooled analysis of three cohort studies
Source: Eur Stroke J. 2026 Aug 24;11(8):aakag100. doi: 10.1093/esj/aakag100 (PMC13502401; doi:10.1093/esj/aakag100)
Supplement: Supplementary file 1 [file supplementary_material_aakag100.zip › Supplemental_Table_9.docx]

**Supplemental Table 9. Kaplan–Meier assessment of risk of recurrent stroke, separately for those with and without TIA as presenting event.**

|  | **Not TIA and CNO without full collapse** | | **Not TIA and CNO with full collapse** | | **TIA and CNO without full collapse** | | **TIA and CNO with full collapse** | |
| --- | --- | --- | --- | --- | --- | --- | --- | --- |
|  | **Events** | **% risk (95%CI)** | **Events** | **% risk (95%CI)** | **Events** | **% risk (95%CI)** | **Events** | **% risk (95%CI)** |
| **Full collapse defined by appearance, n=333^a^** | | | | | | | | |
| Day 0 | 1 | 0.6 (-0.6 - 1.9) | 2 | 2.5 (-0.9 - 5.9) | 2 | 2.8 (-1.0 - 6.6) | 4 | 14.8 (1.4 - 28.2) |
| Day 2 | 4 | 2.7 (0.1 - 5.2) | 4 | 5.1 (0.2 - 9.9) | 3 | 4.2 (-0.4 - 8.8) | 9 | 33.3 (15.6 - 51.1) |
| Day 7 | 9 | 6.2 (2.3 - 10.1) | 6 | 7.7 (1.8 - 13.6) | 6 | 8.9 (2.0 – 15.8) | 9 | 33.3 (15.6 - 51.1) |
| Day 14 | 9 | 6.2 (2.3 - 10.1) | 8 | 10.5 (3.6 - 17.4) | 6 | 8.9 (2.0 – 15.8) | 9 | 33.3 (15.6 - 51.1) |
| Day 30 | 11 | 8.7 (3.6 - 13.8) | 8 | 10.5 (3.6 - 17.4) | 9 | 18.7 (6.5 – 30.9) | 10 | 37.8 (19.2 - 56.4) |
| Day 90 | 13 | 12.2 (5.3 – 19.0) | 9 | 12.3 (4.7 - 19.9) | 12 | 29.8 (14.1 – 45.5) | 10 | 37.8 (19.2 - 56.4) |
| **Full collapse defined by measurement, n=219^b^** | | | | | | | | |
| Day 0 | 0 | 0.0 (0 - 0) | 3 | 6.4 (-0.6 - 13.4) | 2 | 3.9 (-1.4 - 9.2) | 4 | 21.1 (2.7 - 39.4) |
| Day 2 | 3 | 3.0 (-0.3 - 6.3) | 5 | 10.9 (1.9 - 19.9) | 3 | 5.9 (-0.6 - 12.3) | 8 | 42.1 (19.9 - 64.3) |
| Day 7 | 6 | 6.2 (1.4 - 11.0) | 7 | 15.5 (4.9 - 26.1) | 5 | 10.6 (1.8 – 19.4) | 8 | 42.1 (19.9 - 64.3) |
| Day 14 | 6 | 6.2 (1.4 - 11.0) | 9 | 20.5 (8.5 - 32.5) | 5 | 10.6 (1.8 – 19.4) | 8 | 42.1 (19.9 - 64.3) |
| Day 30 | 8 | 11 (3.0 - 18.9) | 9 | 20.5 (8.5 - 32.5) | 7 | 20.8 (5.3 – 36.3) | 9 | 50.4 (26.1 - 74.6) |
| Day 90 | 10 | 17.8 (6.1 - 29.5) | 9 | 20.5 (8.5 - 32.5) | 9 | 33.1 (12.5 – 53.7) | 9 | 50.4 (26.1 - 74.6) |

*CI: Confidence interval. CNO: Carotid near-occlusion. TIA: Transient ischemic attack. a 154 (46%) had not TIA and not full collapse, 80 (24%) had not TIA and full collapse, 72 (22%) had TIA and not full collapse and 27 (8%) had TIA and full collapse. b 102 (47%) had not TIA and not full collapse, 47 (21%) had not TIA and full collapse, 51 (23%) had TIA and not full collapse and 19 (9%) had TIA and full collapse.*
